# Functionally distinct mutations within AcrB underpin antibiotic resistance in different lifestyles

**DOI:** 10.1038/s44259-023-00001-8

**Published:** 2023-05-10

**Authors:** Eleftheria Trampari, Filippo Prischi, Attilio V. Vargiu, Justin Abi-Assaf, Vassiliy N. Bavro, Mark A. Webber

**Affiliations:** 1 Quadram Institute Bioscience, Norwich Research Park, Norwich, Norfolk NR4 7UQ UK; 2 School of Life Sciences, University of Essex, Wivenhoe Park, Colchester, CO4 3SQ UK; 3 Department of Physics, University of Cagliari, S. P. 8, km. 0.700, 09042 Monserrato, Italy; 4 Medical School, University of East Anglia, Norwich Research Park, Norwich, Norfolk NR4 7UA UK

**Keywords:** Experimental evolution, Antimicrobial resistance

## Abstract

Antibiotic resistance is a pressing healthcare challenge and is mediated by various mechanisms, including the active export of drugs via multidrug efflux systems, which prevent drug accumulation within the cell. Here, we studied how *Salmonella* evolved resistance to two key antibiotics, cefotaxime and azithromycin, when grown planktonically or as a biofilm. Resistance to both drugs emerged in both conditions and was associated with different substitutions within the efflux-associated transporter, AcrB. Azithromycin exposure selected for an R717L substitution, while cefotaxime for Q176K. Additional mutations in *ramR* or *envZ* accumulated concurrently with the R717L or Q176K substitutions respectively, resulting in clinical resistance to the selective antibiotics and cross-resistance to other drugs. Structural, genetic, and phenotypic analysis showed the two AcrB substitutions confer their benefits in profoundly different ways. R717L reduces steric barriers associated with transit through the substrate channel 2 of AcrB. Q176K increases binding energy for cefotaxime, improving recognition in the distal binding pocket, resulting in increased efflux efficiency. Finally, we show the R717 substitution is present in isolates recovered around the world.

## Introduction

Antibiotics are crucial for modern medicine, but their introduction and use have resulted in the widespread emergence of antibiotic-resistant bacteria. Bacteria can rapidly adapt to changing environments, and exposure to antibiotics selects for genetic traits that confer resistance, promoting the expansion of resistant mutants^1^. Several important mechanisms of antibiotic resistance have been described, including enzymatic degradation, target modification or bypass, membrane alterations and changes in efflux activity^2^.

Energy-dependent efflux systems are responsible for the export of toxic compounds from the cell to the environment, are found in all bacteria, and act synergistically with other mechanisms of resistance^3^. In Gram-negative bacteria, efflux systems are tripartite transmembrane protein complexes that secrete molecules from the periplasm to the exterior of the cell. The ‘Resistance Nodulation cell Division’ (RND) efflux family is the most important for antibiotic export^4–7^, and RND systems have been shown to determine the basal level of susceptibility of cells to many antimicrobials.

Within the RND family, the Enterobacterial AcrAB-TolC is the best characterised tripartite efflux system and is built around the energised inner membrane H^+^/drug-antiporter AcrB^5^. The functional unit of AcrB is a homotrimer, containing three functionally interdependent protomers, cycling consecutively through loose (L), tight (T) and open (O) conformational states during the efflux cycle in a supposedly cooperative fashion^8,9^. This allosteric “pumping” allows a drug to be acquired from either periplasmic space or the outer leaflet of the inner membrane and passed out of the cell via a conduit produced by the partner outer membrane factor (OMF) and periplasmic adaptor proteins (PAPs)^4,10,11^.

AcrB can export multiple classes of antibiotics, including macrolides, β-lactams, quinolones, rifamycins, tetracyclines, as well as other substrates, including anticancer drugs, bile salts, dyes and solvents^12–17^. This broad substrate specificity is underpinned by the presence of distinct binding pockets within the pump. Drugs of different molecular weights are suggested to be processed in two principal multisite binding pockets, termed the ‘Proximal Binding Pocket’ (PBP) and the ‘Distal Binding Pocket’ (DBP), which have wide specificities and are separated from each other by the so-called gating or switch-loop^8,18–21^. High-molecular-weight drugs appear to be predominantly recognised by the PBP, and recent evidence suggests they may be exported directly to the OMF, bypassing the DBP altogether^22^, whilst low-molecular-weight drugs are thought to be processed predominantly within the DBP^8,19^. Access to these multisite binding pockets is governed by at least four distinct substrate channels, each of which also exhibits different substrate specificities^22–26^. The principal periplasmic drug access channel for polar compounds is proposed to be channel 2 (CH2), preferred by macrolide, rifamycin and tetracycline antibiotics^23,26^, while hydrophobic compounds, such as linezolid, phenicols, fluoroquinolones and novobiocin are suggested to be acquired from the outer leaflet of the inner membrane via channel 1 (CH1). Compounds entering via CH1 and CH2 are thought to pass sequentially through both the PBP and DBP, with access to the latter being restricted by the switch-loop. On the other hand, channel 3 (CH3), implicated in the transport of planar aromatic cations (PACs), such as benzalkonium chloride, crystal violet, ethidium bromide, methylene blue, and rhodamine 6G, is suggested to bypass the PBP and the gating loop altogether, allowing direct access to the DBP^26^. Similarly, membrane-localised carboxylated substrates, such as fusidic acid and hydrophobic β-lactams, access the pump via a groove between the transmembrane helices TM1 and TM2, which forms part of the recently described CH4, again bypassing the PBP, allowing direct access to the DBP^25^.

Whilst AcrB helps determine the intrinsic level of susceptibility to many drugs, it can also confer resistance when overexpressed due to mutations in the regulatory circuits controlling its production^27,28^. Changes within AcrB itself that alter the export of specific antibiotics can also be selected by antibiotic exposure^3,6,29–32^. For example, substitutions M78I and P319L were shown to confer decreased susceptibility to multiple antimicrobial substrates^33^, and substitution G288D has been linked to increased tolerance against ciprofloxacin^29^. These examples demonstrate how selection can favour strains with mutant AcrB proteins altering substrate recognition or export efficiency, as well as mutations in regulators which control pump expression.

Despite the benefits provided, the selection of resistance can have impacts on the fitness of a bacterium, and the fate of any resistance mutation that occurs within a population will depend on how permissive it is for the organism’s lifestyle^34^. Efflux pumps contribute to various important cellular functions, including those relevant to infection. Relationships between efflux pump function and the ability to form biofilms have been established in multiple species^35^, and loss of pump function commonly compromises virulence^36^. Life within a biofilm is common for bacteria and is an important determinant of many infections, as biofilms are also, by nature, highly tolerant of antibiotics^37^.

In this work, we used an evolution model to study how subinhibitory concentrations of two clinically important antibiotics, cefotaxime (Cef) and azithromycin (Azi), representing two major structural classes of antibiotics, cephalosporins and macrolides respectively, selected for resistance mechanisms in *Salmonella*, in both biofilm and planktonic conditions. We found that both antibiotics selected for unique substitutions within AcrB. We confirmed these substitutions affect antibiotic susceptibility and identified their prevalence in the real world of these mutant *acrB* alleles. Using structural and computational approaches supported by genetic and phenotypic analysis, we demonstrate how these two distinct substitutions within AcrB facilitate drug translocation through the efflux conduit of the pump in fundamentally different ways.

## Results

### Cefotaxime and azithromycin both select for substitutions within AcrB

To investigate the adaptation of *Salmonella* to clinically important antibiotics, we used representatives of two antibiotic families amongst the drugs of choice for the treatment of salmonellosis: cefotaxime, a third-generation cephalosporin, and azithromycin, a second-generation macrolide. We repeatedly exposed independent planktonic and biofilm lineages of *S*. Typhimurium 14028S to concentrations of azithromycin and cefotaxime that restricted planktonic growth rates by ~50% (10 and 0.062 μg/ml, respectively) for 17 passage cycles (each lasting 72 h). Estimation of the number of generations each population went through (based on calculating log2 × the dilution factor of cells in each condition by the number of passages) gave ~170 for planktonic conditions, ~264 for cefotaxime-exposed biofilms, ~289 for azithromycin-exposed biofilms and ~317 for control biofilms. The number of generations was higher for biofilms than planktonic conditions as we used a bead-based evolution model^38^, where the dilution factor of cells which occurs when new, sterile beads are colonised, is higher than the dilution in planktonic cultures.

Phenotyping of isolates recovered over time from the experiments found that both antibiotics rapidly selected for resistance (Supplementary Fig. 1). Genome sequencing identified drug-specific mutations resulting in substitutions within AcrB. Cefotaxime selected for a Q176K substitution, and azithromycin for an R717L substitution. To define the phenotypic impacts of these mutations in more detail and to determine when they emerged in each experiment, three single colonies were recovered from each of three time points (early, middle and late; corresponding to passages 1, 9 and 17, respectively). Isolation of single isolates was carried out for each of the four independent exposed biofilm lineages, as well as the exposed planktonic and unexposed biofilm control (20 isolates in total, derived from exposed conditions). These mutants were then phenotyped and genome sequenced.

**Fig. 1:**
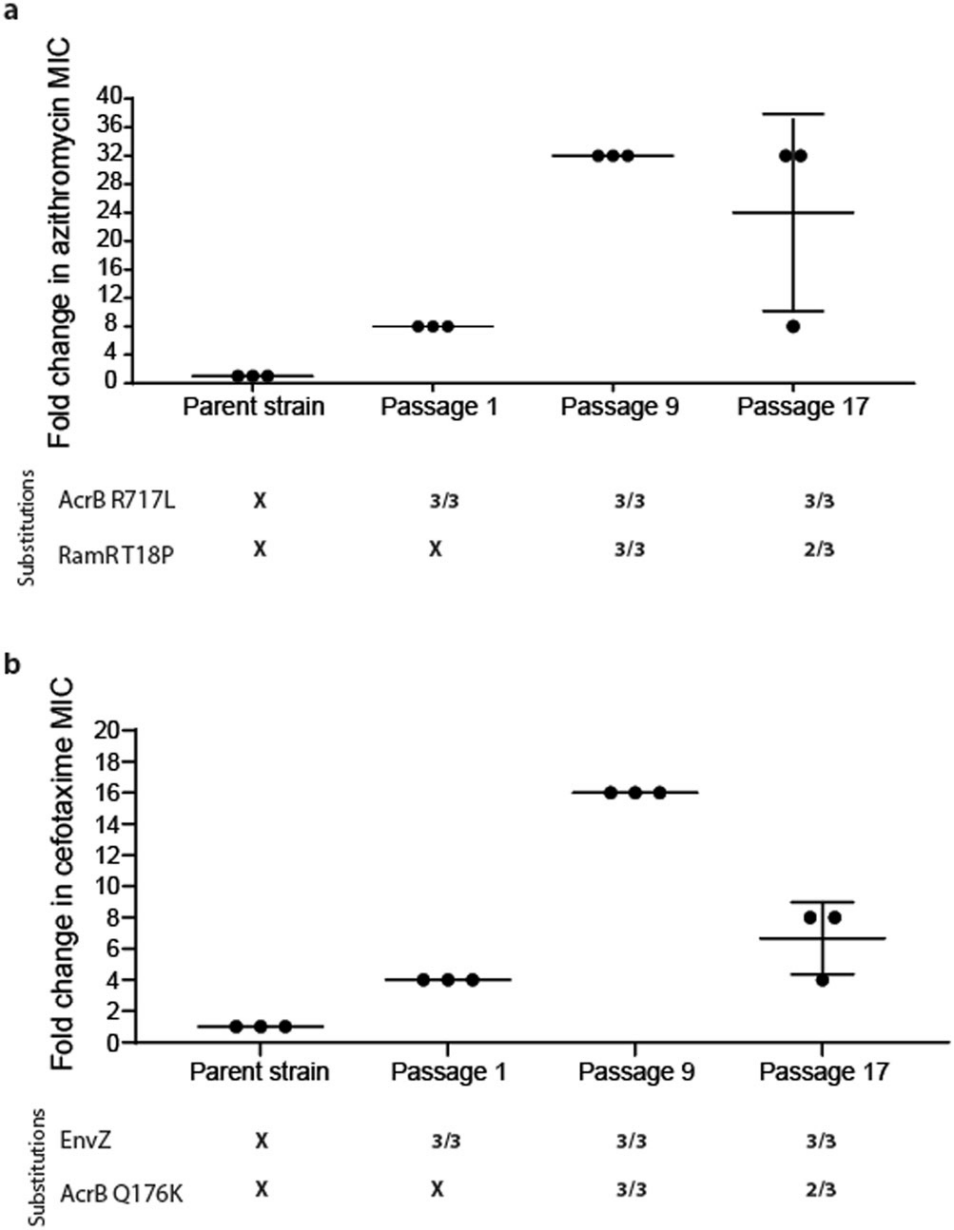
Selection of substitutions within AcrB in different conditions. **a** Azithromycin selection. Three isolates were phenotyped and sequenced from biofilms passaged 1, 9 and 17 times, respectively. AcrB R717L emerged after passage 1 and led to an eightfold increase in azithromycin MIC. Five isolates (out of the six) from passages 9 and 17 also carried an additional RamR T18P substitution conferring a fourfold additional increase in azithromycin MIC. **b** Cefotaxime selection. Three isolates from a planktonic population were phenotyped and sequenced after passages 1, 9 and 17. Mutations within *env*Z emerged after passage 1, conferring fourfold increase in MIC. By passage 9, the AcrB Q176K substitution emerged, which led to a 16-fold change in MIC. Isolates from passage 17 exhibited a four- to eightfold change in MIC. Any MIC change of twofold or above was considered significant. Long horizontal bars indicate the average value for each condition and smaller error bars the standard deviation.

Exposure to azithromycin rapidly selected for the R717L mutation within AcrB after just a single exposure under stress in all populations regardless of the selective context (biofilm or planktonic). The R717L mutation was associated with an eightfold increase in the minimum inhibitory concentration (MIC) for azithromycin. Figure 1a shows this substitution was present in all isolates over time from one randomly selected biofilm lineage (as well as being in all the populations sequenced). An additional mutation within the local transcriptional repressor *ramR* controlling the expression of the *acrAB* multidrug operon^39^ (corresponding to a T18P substitution) emerged after passage 9 in addition to the *acrB* mutation. This was associated with a further increase in MIC of azithromycin to 32-fold higher relative to the parent strain. This mutation was also linked with increased MICs of different classes of antibiotics, including chloramphenicol (eightfold increase) and ciprofloxacin (eightfold change), consistent with previous work^40^. No other additional mutations were identified in the isolated mutants, and none were seen to occur repeatedly in multiple populations.

The dynamics of selection for substitutions within AcrB by cefotaxime were different. Initial populations obtained a mutation within *envZ* (R397H), leading to reduced permeability to cefotaxime (which we have recently described in detail in ref. ^41^). In contrast to the azithromycin exposure where the *acrB* mutation emerged first, the Q176K substitution within AcrB emerged halfway through the experiment (passage 9) and was always seen in conjunction with the *envZ* mutation. Notably, Q176K was only recovered from planktonic populations. The acquisition of these two mutations was associated with an MIC increase for cefotaxime to the clinical breakpoint (2 μg/ml), compared to the parent strain’s MIC (0.125 μg/ml) (Fig. 1b). Increased tolerance was maintained throughout the course of the experiment for mutants carrying both substitutions. In passage 17, the measured susceptibility of these strains was a fold lower compared to passage 9. This is not considered significant and is accepted as an error of the method. Fitness, in the form of bacterial growth in liquid culture, of isolates carrying the two identified substitutions, was not affected, as measured by growth curve assays (Supplementary Fig. 2). However, a negative effect on biofilm formation was observed.

### Characterisation of the role of AcrB substitutions in resistance

To confirm the changes observed within AcrB were responsible for the decreases in susceptibility observed for the corresponding selective drugs, we recreated the relevant genotypes in the parent *Salmonella* strain. We then determined their impact on sensitivity to a panel of drugs and on cellular permeability to the efflux substrate, resazurin.

We generated a mutant of the parent strain 14028S lacking *acrB* and complemented it with either wild-type or mutant alleles on a plasmid to determine the impacts on phenotypes observed (Table 1A). The introduction of AcrB R717L to the *ΔacrB* background led to resistance against azithromycin only, matching the phenotype of the adapted strains carrying the AcrB R717L mutation. The additional introduction of RamR T18P led not only to a further increase in MIC of azithromycin but also to MICs of chloramphenicol, nalidixic acid and tetracycline, showing that this substitution does not compromise other substrates and that the overexpression of the efflux pump is the major determinant for MDR (Table 1A).

**Table 1:** Reconstitution of *acrB* genotypes confirms impacts on susceptibility.

**A**						
	**MIC (µg/ml)**					
	**WT**	**AcrB R717L**	**AcrB R717L RamR T18P**	***ΔacrB***	***ΔacrB*** **p*acrB***	***ΔacrB*** **p*acrB*_R717L**
Azithromycin	4	**32**	**64**	*0.5*	4	**16**
Cefotaxime	0.125	0.06	0.125	*0.015*	0.125	0.125
Chloramphenicol	4	8	**16**	*0.5*	4	4
Ciprofloxacin	0.03	0.03	0.06	0.03	0.015	0.03
Kanamycin	4	4	2	4	4	4
Nalidixic acid	2	2	**8**	*0.5*	2	2
Tetracycline	0.5	0.5	**2**	*0.125*	0.5	0.5
	***ΔramR***	***ΔramR*** **p*ramR***	***ΔramR*** **p*ramR_T18P***	***ΔacrB, ΔramR***	***ΔacrB, ΔramR*** **p*acrB***	***ΔacrB, ΔramR*** **p*acrB_R717L***
Azithromycin	**16**	4	**16**	*0.5*	4	**16**
Cefotaxime	0.25	0.06	0.25	*0.015*	0.25	0.125
Chloramphenicol	**16**	4	**16**	*0.5*	8	8
Ciprofloxacin	0.06	0.03	0.06	0.03	0.03	0.06
Kanamycin	4	ND	ND	2	4	4
Nalidixic acid	**8**	2	**8**	*0.5*	4	4
Tetracycline	**2**	0.5	**2**	*0.125*	1	1

**B**						
	**WT**	**EnvZ R397H**	**EnvZ R397H AcrB Q176K**	***ΔenvZ***	***ΔenvZ*** **p*envZ***	***ΔenvZ*** **p*envZ_R397H***
Azithromycin	4	4	2	4	4	8
Cefotaxime	0.125	**0.5**	**1**	0.125	0.125	**0.5**
Chloramphenicol	4	**16**	**16**	8	ND	**16**
Ciprofloxacin	0.03	0.06	0.03	0.03	0.03	0.06
Kanamycin	4	2	4	4	ND	ND
Nalidixic acid	2	4	4	2	2	4
Tetracycline	0.5	1	**2**	0.5	0.5	1
	***ΔacrB***	***ΔacrB*** **p*acrB***	***ΔacrB*** **p*acrB_Q176K***	***ΔacrB, ΔramR***	***ΔacrB, ΔramR*** **p*acrB***	***ΔacrB, ΔramR*** **p*acrB_Q176K***
Azithromycin	*0.5*	2	2	*0.5*	4	2
Cefotaxime	*0.015*	0.125	0.125	*0.015*	0.25	**1**
Chloramphenicol	*0.5*	4	**8**	*0.5*	8	**16**
Ciprofloxacin	0.03	0.015	0.015	0.03	0.03	0.03
Kanamycin	4	4	4	2	4	4
Nalidixic acid	*0.5*	2	2	*0.5*	4	4
Tetracycline	*0.125*	0.5	1	*0.125*	1	**2**

**A** Complementation of AcrB R717L in *ΔacrB* and of RamR T18P in *ΔramR* background reproduced the resistance profiles of the strains isolated from the evolution experiments, confirming that these substitutions are key to the resistant phenotypes observed. **B** Complementation of Q176K in the *ΔacrB* background had no pronounced impact on cefotaxime resistance until combined with either *ΔramR* or EnvZ R397H, where it then conferred decreased susceptibility to cefotaxime, chloramphenicol, and tetracycline causing an MDR phenotype. ND indicates not determined due to the presence of confounding resistance cassettes. Values in bold indicate a fourfold or higher increase in MIC compared to the WT, and those in italics a fourfold or higher decrease.

While the complementation of the *acrB* deletion strain with *acrB*-Q176K did not have a detectable impact on cefotaxime resistance (Table 1B), the complementation of *acrB* in a Δ*acrB*/Δ*ramR* background (which results in overexpression of *acrB* due to loss of RamR, and hence make the impact of the complementation clearer) with the *acrB*-Q176K allele did replicate the phenotype of strains derived from the evolution experiments. Similarly, a strain with chromosomal mutations conferring both AcrB Q176K and EnvZ R397H also showed an MIC of cefotaxime fourfold higher than the parent strain. These data confirmed the specific role of AcrB Q176K in cefotaxime sensitivity but also showed that a significant change in MIC requires synergistic mutations in either *ramR* or *envZ*.

### Impact of substitutions on efflux substrate accumulation and gene expression

**Fig. 2:**
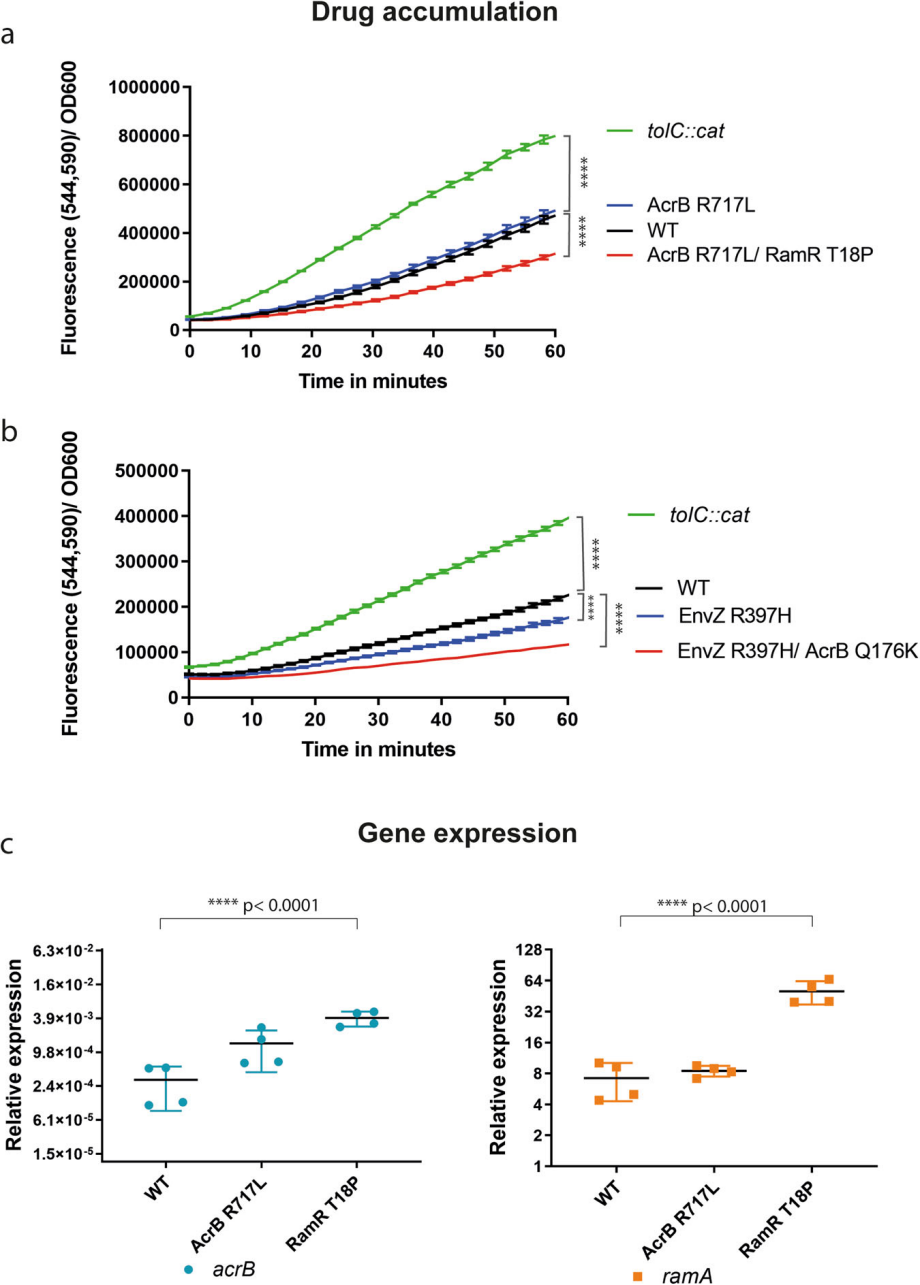
Accumulation of the efflux substrate resazurin and expression of efflux genes. **a** Reduced accumulation was observed in strains carrying both AcrB R717L and RamR T18P substitutions (*p* < 0.0001). **b** Mutants carrying EnvZR397H exhibited decreased drug accumulation. Additional mutation within AcrB (Q176K) led to a greater reduction in the accumulation of resazurin in the cells (*p* < 0.0001). *tolC::cat*, pump-defective mutant, was used as a control. **c** qRT-PCR in 48-h biofilms. Expression of *acrB* and *ramA* was monitored in an isolate carrying the AcrB R717L substitution and in a strain carrying both the AcrB R717L and the additional RamR T18P. The increase of expression of *acrB* and *ramA* was significantly higher compared to the WT in the presence of the RamR T18P substitution. Error bars reflect estimates ± one standard error. Statistical significance was calculated using a two-way ANOVA test.

To further confirm whether the Q176K and R717L AcrB substitutions altered general drug accumulation or efflux activity, we monitored the intracellular accumulation of resazurin^42^ (Fig. 2). Resazurin is a non-fluorescent dye which, upon cell entry, undergoes a redox reaction leading to colour change. We used WT (14028S) as our reference and a *tolC*-deficient mutant as a control lacking functional efflux. The R717L mutant alone did not show any changes in resazurin accumulation compared to the WT, suggesting the substitution does not impact the export of this substrate (Fig. 2a).

The AcrB Q176K substitution was always present in strains already carrying the EnvZ R397H substitution. We measured resazurin accumulation in strains carrying only the EnvZ substitution and strains also carrying the additional AcrB Q176K substitution. Mutants carrying EnvZ R397H alone accumulated less resazurin compared to the WT, and the addition of the AcrB Q176K substitution resulted in a further decrease in resazurin accumulation, consistent with an increase in efflux efficiency (Fig. 2b).

To further confirm the role of the RamR substitution seen under azithromycin exposure on pump expression, we extracted RNA from 48-h-old biofilms, and we measured the expression of *acrB* and *ramA* by qRT-PCR, using *gyrB* expression as our internal reference (Fig. 2c). Both genes were found to be derepressed in the mutants compared to the parent strain.

### In silico modelling reveals a distinct role of R717L substitution in the substrate specificity of the pump

**Fig. 3:**
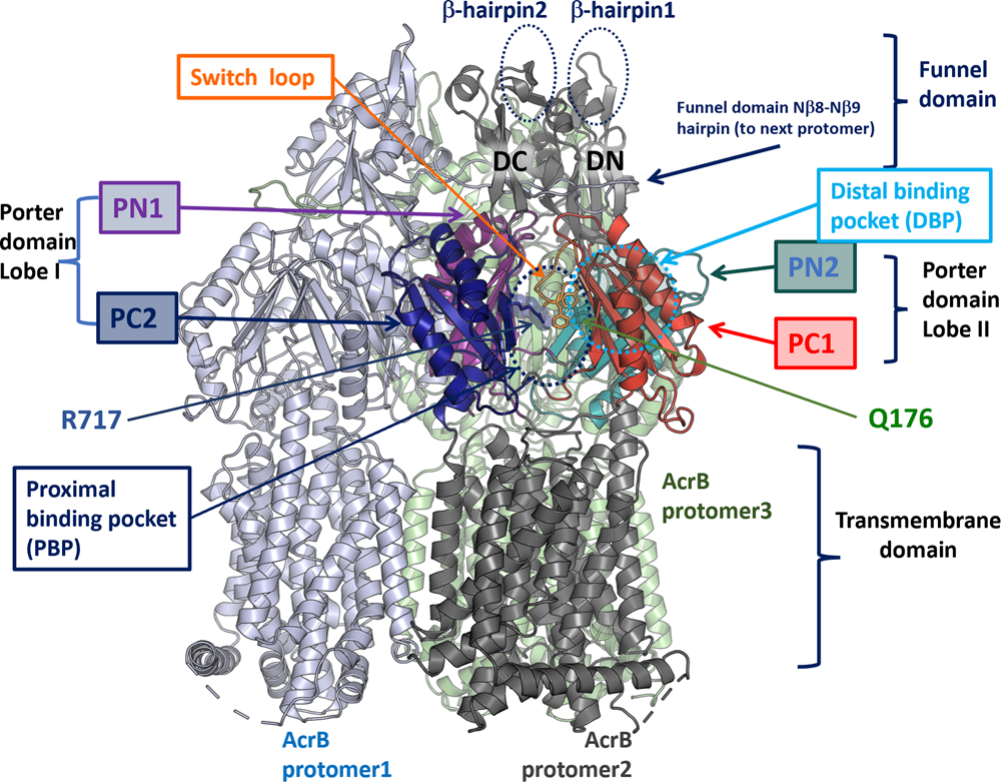
Structural organisation of the AcrB trimer indicating the location of mutated residues with relevant substitutions and their relation to the proximal and distal binding pockets. A single protomer (protomer 2) is annotated, with transmembrane helices and the funnel domain in dark grey, while the porter domain subdomains (PN1, PC1, PN2 and PC2), which form the main substrate recognition channels and drug-binding pockets are colour coded. Approximate locations of the PBP and DBP are given with dotted circles. The sidechains of R717 and Q176 are shown as sticks. The switch-loop, separating the PBP from DBP, is coloured in orange, and the conserved residues F615 and F617, which belong to the loop, are also shown as sticks for reference. Illustration based on the experimental structure of the *S*TmAcrB 6Z12.PDB^43^.

Analysis of the 3D structure of *Salmonella* Typhimurium AcrB (*S*TmAcrB)^43^ indicated that both the acquired substitutions map within the multisite drug-binding pockets of the transporter, with R717L occupying the front end of the PBP, close to the exit of the substrate channel CH2, and Q176K being located in the DBP (Fig. 3), suggesting that they may impact drug interaction directly and specifically, rather than having a general or allosteric effect. To gain further mechanistic insight into their effect, we performed in silico docking of the respective antibiotics to both WT and mutationally modified drug-binding pockets of *S*TmAcrB.

To enable docking, we needed to identify suitable docking templates based on both the ligand occupancy and the functional state of the transporter. The only available experimental structure of *S*TmAcrB (PDB ID: 6Z12)^43^ is an apo-structure derived from cryo-electron microscopy at a modest resolution (4.6 Å), making accurate side chain predictions within the respective binding pockets unreliable. Furthermore, the structure is C3-symmetrised, and hence binding pockets could not be assigned to either of the physiologically relevant L, T or O-conformations, making that structure poorly suited for the intended docking studies. Fortuitously, the multisite drug-binding pockets of *Salmonella* and *E. coli* AcrB are highly conserved, with only three substitutions, namely S48T, E280K and M573L, affecting the lining of the drug-binding pockets. Of these, only M573 is predicted to participate in the binding of macrolide and rifampin-like compounds within the PBP according to the available crystal structures^19,22^, while E280K (which is only participating in the formation of the pocket via its main-chain atoms), and the conservative S48T substitution, might have a limited effect in the DBP^19,21^. Taking these considerations into account and following the previous protocol^44^, we performed ensemble docking of azithromycin and cefotaxime onto the DBP, PBP and CH2 entrance channel (that is, the sites containing the mutated residues) of several homology models of the *Salmonella* AcrB derived from the available high-resolution X-ray crystal structures of the *E. coli* orthologue, which present the functionally relevant ligand-bound L- and T-conformers^19,45^ (see “Methods” for details). For each ligand and each binding site, the top docking pose was further relaxed, as this has been shown to improve accuracy^46^.

**Fig. 4:**
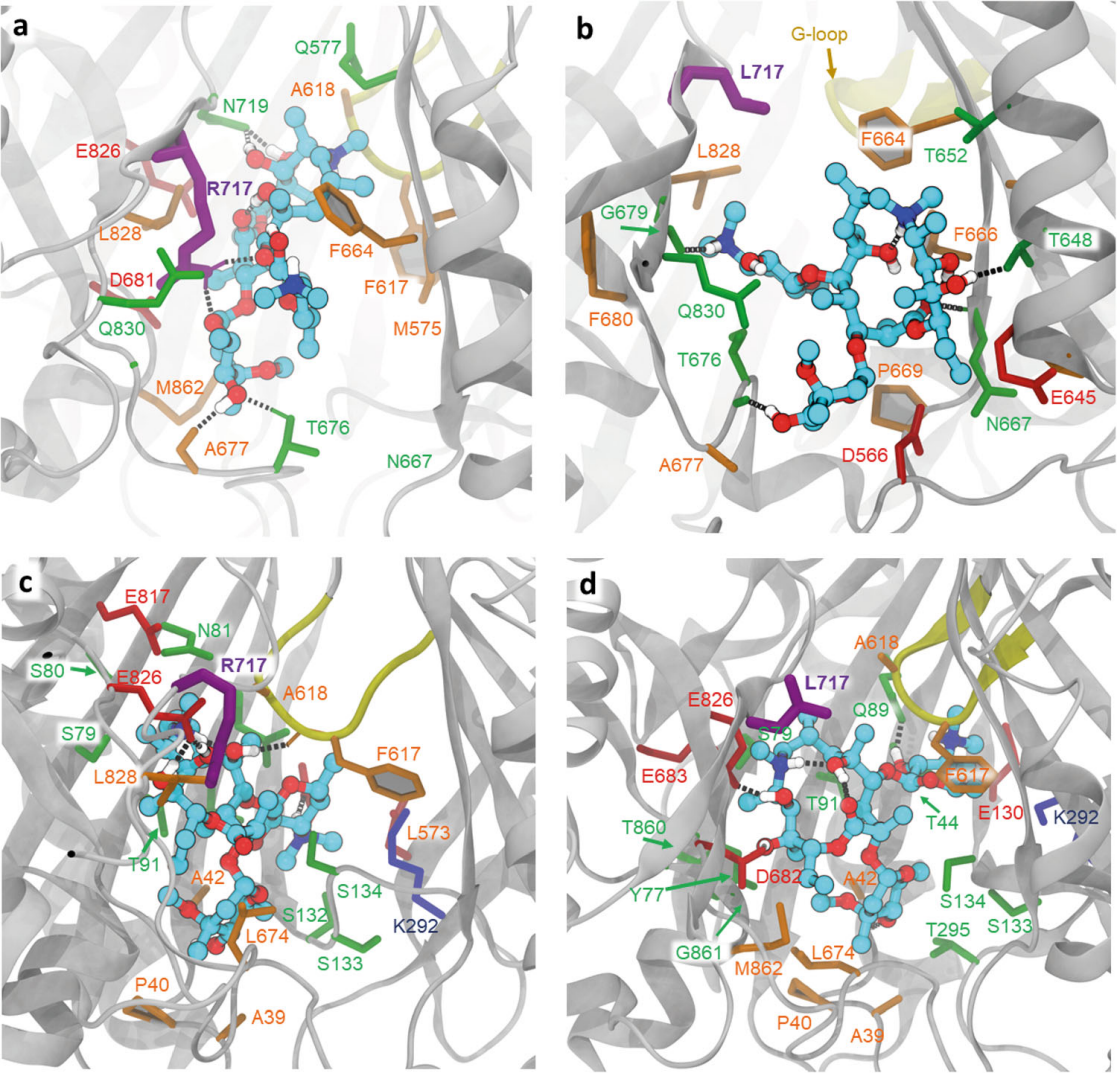
Docking of Azithromycin to the entrance of CH2 and PBP. All residues within 2.5 Å of the docked ligands (plus the residue R/L717) are shown in stick representation. **a** Relaxed top pose of azithromycin bound to the entrance of CH2 in the WT, showing ligand coordination with the participation of R717 (purple thick sticks). Dotted lines represent polar contacts. Additional charged (red) and polar (green) residues providing essential contacts are T676, D681, N719 and Q830, as well as the hydrophobic F664, L828 and M862 (in orange). **b** The CH2 entrance in the R717L variant shows radically different coordination of the ligand, as it slips towards CH2, losing contact with L717(purple) and forming new contacts on the opposite side of the channel—e.g., F666 and P669. **c** Relaxed top pose for azithromycin bound to the PBP. The R717 does not participate in the coordination of the azithromycin. Note the participation of E826 and the gating-loop residues F617 and A618 in coordination. **d** Relaxed top pose for azithromycin bound to the R717L PBP, showing minor adjustment of coordination, with the participation of the gating loop and involvement of Q89.

We first focused our attention on the R717L substitution and performed ensemble docking of azithromycin (abbreviated to Azi below). We performed two separate runs, one centred at the PBP and the second centred at the CH2 access channel of AcrB. When centring the docking grid on CH2, the top poses in the WT cluster closely together (Supplementary Fig. 3) and overlap with the site that is involved in substrate binding observed in the L-protomer rifampicin/3-formyl rifamycin SV-bound structures^19,22^, but not macrolide-bound structures. Intriguingly, the top WT docking pose for Azi shows direct involvement of R717 (alongside neighbouring residues N719, L828 and Q830) in ligand coordination (Fig. 4a), which is consistent with residue contacts seen in rifampicin/3-formyl rifamycin SV/rifabutin, but not macrolide-occupied crystal structures.

In the case of the R717L mutant, the poses also cluster tightly together; however, they centre closer to the front end of the PBP, overlapping the CH2 exit (Supplementary Fig. 3). Correspondingly, the R717L mutation resulted in radically different coordination of Azi from the one observed in the WT (Fig. 4b), and loses contact not only with the R717L itself but also its polar contacts with D681, N719 and E826. While Q830 is still providing coordination, several hydrophobic contacts are created from the opposite side of the pocket, notably F664, F666 and P669.

Supporting the idea that the preferred CH2 binding site of Azi diverges in the R717L mutant when compared to the WT, the top pose of binding of Azi to CH2 in the mutant R717L structure has a significantly lower binding score (~2 kcal/mol, Table 2), than in the WT protein.

**Table 2:** Pseudo-free energy of binding of top poses of azithromycin for the two ensemble docking runs in the CH2 and PBP after relaxation.

Top poses from ensemble docking	Centre on CH2 kcal/mol	Centre on PBP kcal/mol
WT	−11.8	−13.9
R717L	−10.0	−13.9

These different affinities can be rationalised by a change of coordination, as while in the R717L-pocket, the top pose includes additional coordination with the participation of Q830 and retains L728, it loses the essential N719, E826 and L717 contacts. Taken together, this suggests that azithromycin features different binding modes to the WT and R717L, with more stable contacts with CH2 in the WT form, which may translate into lower residence times for it in the case of R717L.

After entry via CH2, Azi is thought to move into the PBP, where its primary binding site is located, as demonstrated by several macrolide-AcrB structures^19,22,47^. In agreement with that, when docked at the centre of the PBP (Fig. 4c, d), Azi preferentially clusters into the back of this site in both WT and R717L structures. These Azi docking positions broadly overlap with the observed substrate position in the erythromycin-occupied experimental structures^19,22,47^ and notably are associated with loss of contact with R/L717. The pseudo binding free energies of the top poses of this compound to the PBP are very similar in both the WT and R717L variants of AcrB (Table 2), consistent with our interpretation that the enhanced efflux of Azi seen in the R717L mutant is due to changes in CH2 rather than altered coordination within the PBP itself.

Our docking results suggested that the R717L substitution would mostly impact substrates relying on PBP sequestering and entering the PBP via CH2 (e.g., macrolides, rifamycins and other ansamycins). Anthracyclines such as doxorubicin and tetracycline antibiotics are also thought to utilise CH2, but appear to bypass PBP altogether and are instead sequestered directly in the DBP^22,45,48^, so R717L would be expected to have a smaller impact on their efflux. Finally, substrates that enter the PBP via the membrane-linked CH1 (including linezolid, fusidic acid, and novobiocin) and planar cations, such as EtBr that are thought to enter directly into DBP via CH3^22,26^, are expected to be relatively unaffected by the R717L. To challenge these predictions, the susceptibility of defined mutants to members of the above compound classes was tested. Consistent with our hypothesis, the MICs of the other tested macrolides and rifampicin were similarly affected, while tetracycline, doxorubicin and novobiocin showed no significant differences, and linezolid was unaffected by the R717L substitution (Table 3).

**Table 3:** MICs of compounds which do not utilise CH2 are not affected by R717L.

MIC(μg/ml)	Azi	Ery	Cla	Tet	Rif	Lin	Nov	Dox
14028S (WT)	8	64	64	1	12	256	200	200
14028S ΔAcrB	1	2	2	0.25	6	8	3.125	1.56
ΔΑcrB/ pWKS30-pacrB_WT	8	64	32	0.5	6	128	100	200
ΔΑcrB/pWKS30-pacrB_R717L	**64**	**256**	**256**	0.5	6	128	50	200

Results show the mean of three independent experiments. Bold values indicate significant changes.

*Azi* azithromycin, *Ery* erythromycin, *Cla* clarithromycin, *Tet* tetracycline, *Rif* rifampicin, *Lin* linezolid, *Nov* novobiocin, *Dox* doxorubicin.

To extend these observations beyond Azi, we conducted additional single-structure docking using AutoDock Vina, using structures PDB 3AOC and 3AOB. The preferential binding mode for most tested compounds appears to be within the back part of the PBP, which, consistent with our predictions, appears to be undisturbed by the mutation. The only notable exceptions are for Cla and Ery, which appear to form novel hydrophobic interactions in the front part of the PBP in the case of R717L. That also coincides with a loss of interaction of these compounds with the R717 side chain and might help explain the observed differences in the MIC (data not shown).

### In silico modelling predicts AcrB Q176K affects substrate recognition in a distinct manner to R717L

To investigate the impact of the Q176K substitution on the *S*TmAcrB structure and substrate binding, we performed in silico modelling of the distal binding pocket of the *S*TmAcrB using homology models of the *Salmonella* DBP based on the experimental *E. coli* structures, followed by ensemble docking of cefotaxime (Cef) as described above for the PBP (Supplementary Fig. 4).

**Fig. 5:**
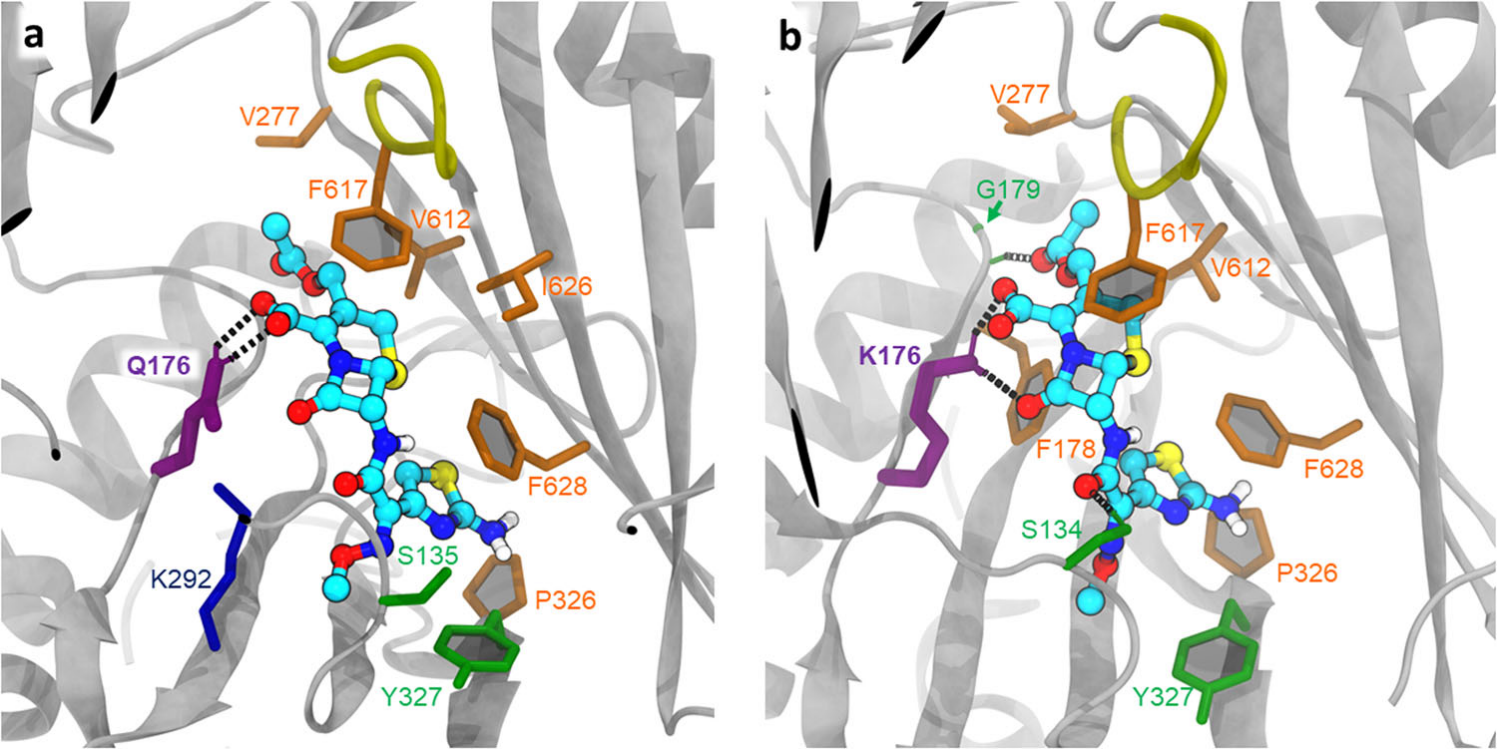
Effect of Q176 substitution on the coordination of cefotaxime in the deep binding pocket from ensemble docking studies. **a** Relaxed top pose coordination showing the essential residues in the WT. Side chain of the Q176 (thick purple sticks) directly participates in ligand binding, providing polar contacts; ligand binding is additionally supported by predominantly hydrophobic interactions (orange). **b** Relaxed top pose for Q176K, demonstrating the increased coordination with the participation of K176. S135 and G179 (via main chain) provide additional polar contacts (green); however, overall, the position of the Cef in the DBP remains nearly identical to the one observed in the WT.

The best poses found for Cef in the DBP of the T monomer (after structural relaxation) are shown in Fig. 5. The corresponding observed binding score is −8.4 and −9.7 kcal/mol for the WT and Q176K, respectively, which is opposite to the situation observed with R717L and Azi binding to the CH2. Here, the introduction of the Lys-residue into the DBP results in a direct increase of hydrogen bonds between the protein and the ligand (Fig. 5b), which translates into a better fit for the drug and correspondingly higher energy of binding. This suggests that the mechanism by which the Q176K substitution aids Cef export is radically different from that by which R717L substitution affects Azi efflux.

We corroborated these docking results by additional single-structure docking of the related compounds—cephalothin and nitrocefin, both of which showed very limited displacement but a notable change of coordination with the addition of Q176K (data not shown).

### Differential abundance of AcrB substitutions in globally dispersed isolates

**Fig. 6:**
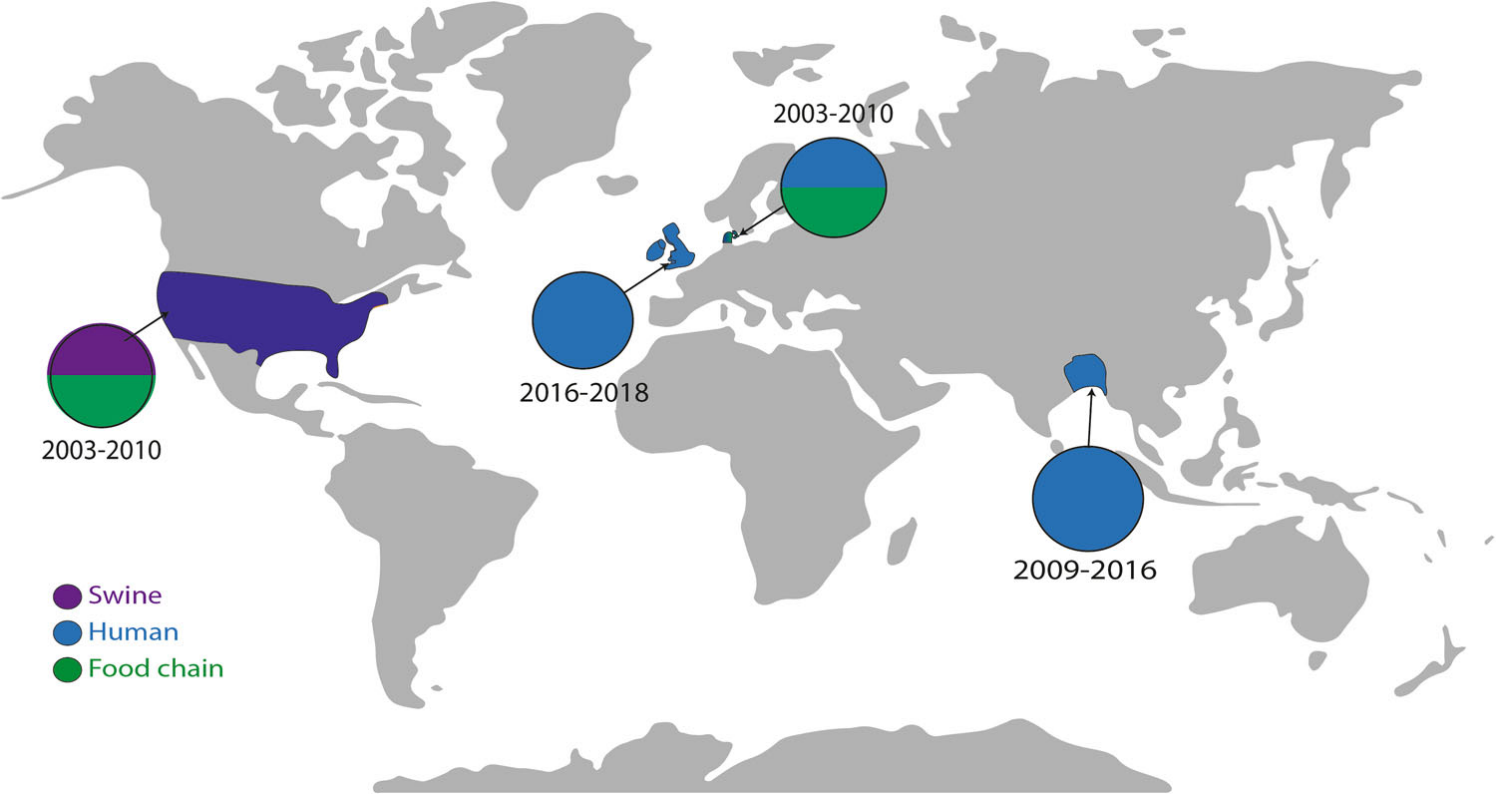
Identification of R717L in geographically diverse isolates. The map shows where isolates carrying AcrB with the R717L substitution have been reported. Isolates from swine are indicated by purple, clinical isolates with blue, and isolates from the food chain are highlighted in green. Isolates of *S*. Typhimurium were isolated from the United States, United Kingdom, Ireland and Denmark. Clinical isolates of *S*. Typhi resistant to azithromycin were recorded in Bangladesh^52^. Isolates carrying the R717L allele were isolated between 2003 and 2019.

To determine whether the mutations selected in this study were biologically permissive and in circulation in the real world, we searched for their presence in EnteroBase, which contains over 200,000 *Salmonella* genomes deposited from around the globe^49,50^. Whilst we first reported the AcrB R717L allele in 2019^51^, a search of the deposited strains identified it in 12 *S*. Typhimurium isolates originating from patients, livestock and food in the United Kingdom, United States, Ireland and Denmark, with the first deposition being in 2003 (Fig. 6). A recent study also identified substitution at R717 in multiple azithromycin-resistant isolates of *S*. Typhi (R717Q) and Paratyphi A (R717L) from patients in Bangladesh^52^. These findings demonstrate that this substitution has been selected on multiple occasions in different *Salmonella* serotypes around the world. The Q176 substitution was not identified in the database.

## Discussion

Antibiotic resistance is a complex phenomenon, and it has become clear that the physiological state of bacteria has a large impact on resistance. Recent work has focused on how biofilms can evolve resistance and has shown that for some species, there are biofilm-specific routes to resistance or that developing resistance can affect biofilm formation itself^38,41,53^. In this study, we identified sub-inhibitory concentrations of two critical antibiotics rapidly selected for substitutions within AcrB as a central mechanism underpinning the evolution of resistance of *Salmonella* both in planktonic and biofilm states. Adaptive mutations of RND pump proteins are being increasingly reported and represent a general frontline mechanism of bacterial response to antibiotic and other environmental stress^33,54,55^. However, there is little current understanding of how the various changes reported act mechanistically and what impacts there may be on the capacity of the pump to export other substrates. In this work, we characterise two substitutions in detail, which allows their mechanisms to be understood and demonstrates two fundamentally different modes of action. The importance of the two mutations in the biology of the cell also appears to differ, which may reflect their relative importance in the real world.

One of the first AcrB-specific mutations to be isolated due to antibiotic treatment in a clinical setting resulted in a G288D substitution in *Salmonella* AcrB^29^. This conferred clinically significant ciprofloxacin resistance isolated from an infection which proved fatal to the patient. Additional M78I and P319L substitutions within AcrB have also been identified in ciprofloxacin-resistant isolates of *Salmonella*^33^. Substitutions have also been reported within AcrB, which confer resistance in *Klebsiella*^54^, as well as in the related CmeB RND transporter in *Campylobacter*^55^.

R717 is located on the upper side of the access CH2 exit and contributes to the formation of the frontal part of the PBP, where it can directly coordinate rifampicin^19^, 3-formyl rifamycin^22^ and a number of smaller compounds (e.g., ciprofloxacin^56^ and doxorubicin^45^ in the L-conformers of *E. coli* AcrB. As revealed by a number of experimental structures, R717 is the focus of a multi-residue network, including the side chain of Q830 and backbone atoms of S715 and L828, involved in the coordination of rifampicin and 3-formyl rifamycin. While R717 is not seen directly interacting with erythromycin molecules in the PBP of the available structures (e.g., PBD ID 3AOC^19^;), it is within interacting distance of other critically important ligand-coordinating residues such as N719, which can provide direct bonding both with the erythromycin substrate alongside E826 (e.g., PDB ID 4ZJQ^47^).

Thus, the substitution of R717 with a hydrophobic, bulky leucine residue could be expected to influence efflux efficiency via a direct change in drug coordination, as well as via secondary effects, due to disruption of the charged residue networks and general changes in the electrostatics and solvation in the pocket. While short of a direct experimental validation, our ensemble docking results support these predictions. Docking of azithromycin to the CH2 entrance of the WT protein resulted in a tight clustering of high affinity poses in the proximity of R717 (Supplementary Fig. 3), with the top pose making extensive direct contact with the side chain of this amino acid (Fig. 4a). This coordination is not directly observed in the available erythromycin-bound structures, but is highly compatible with the rifampicin, 3-formyl rifamycin, and rifabutin-bound structures, e.g., PDB IDs 3AOB; 6ZOB; 6ZO9^19,22^, and we propose that such a pose represents a valid transient interaction of the macrolide ligands during their transit from the CH2 channel into the PBP proper. The predicted interaction can also readily explain the observed impact of R717 on MICs of both macrolide and ansamycin antibiotics that we observed (Table 3). Consistent with this interpretation is the dramatic change of coordination we observed when docking azithromycin to the R717L pocket, resulting in an unexpected shift, or “slippage” of the preferred azithromycin docking positions down towards the bottom of CH2 (Supplementary Fig. 3, Fig. 4b). This loss of coordination with several residues participating in the stabilisation of the ligand in the WT translates into a significant difference in the estimated binding energy of azithromycin to the R717L pocket. This observation provides strong evidence for a structural impact on CH2 impacting azithromycin transit. However, we also wanted to explore any possible impact on the second canonical macrolide binding site within the PBP. There, the preferred docking poses for both WT and R717L overlap and align with experimental macrolide-bound structures^19,22,47^ (Fig. 4c, d). This is expected, given that this binding site does not allow direct contact of the ligand with either R717 or L717 side chain, and correspondingly there is no measurable difference in the pseudo binding free energy of azithromycin to this site (Table 2). These data are important as they suggest that while the recognition and energy of binding in the back of PBP is not affected by R717L substitution, the mutation has a dramatic impact on the front of the ligand transport pathway (CH2) associated with the initial stages of macrolide/ansamycin transport. Previously, stepwise transfer of substrates through the efflux duct of AcrB has been suggested by the available substrate-occupied X-ray and cryo-EM structures of AcrB^9,19,45,57^, as well as by a number of molecular dynamics simulations^48,58,59^ and our in silico data strongly support these predictions.

Taken together, our analysis suggests that while the R717L mutation affects access to CH2 by the large macrolide compounds, it does not affect the PBP’s affinity towards these classes of drugs. This was further supported by the differential impacts that the R717L mutation had on drugs predicted to utilise different substrate channels (Table 3). Indeed, the observed 2 kcal/mol differences in binding energies between the WT and R717L in the front of the CH2, but not in the back of the PBP, suggests that the retention time of drugs such as azithromycin might be lower in the mutant, facilitating the drug transition from the CH2 to the back of the PBP, without impacting recognition in the latter. This is important, as it could explain how this substitution does not result in loss of ability to export other AcrB substrates and so does not prevent the MDR phenotype observed when R717L was overexpressed.

Subsequent to our first description of R717L^51^, a recent study by Zwama and Nishino^60^ has provided evidence which indicated steric hindrance and electrostatic effects to be the cause of a change in the relative accessibility of the PBP. This supports the work we report here, and we now significantly expand the scope of that study by providing a quantitative assessment of drug binding and the specific molecular environment within the binding pockets of the pump to further understand the molecular mechanisms of this mutation.

The importance of changes at R717 (*Salmonella* AcrB numbering) is further supported by a recent report of mutations in the orthologous Neisserial transporter MtrD, associated with increased azithromycin MICs—namely R714G and K823E substitutions^61,62^. This led the authors to speculate that non-mosaic gonococcal strains bearing both the *mtrR* promoter and amino acid changes at MtrD positions 714 or 823 could translate into clinically significant levels of azithromycin resistance. A follow-up study using a global meta-analysis collection of 4852 *Neisseria gonorrhoeae* genomes^62^ did identify the residue R714 of MtrD as a hotspot for mutations leading to increased MICs against azithromycin arising in clinical settings. Several alleles of R714 have been reported from clinical isolates, including R714L, as well as R714C and R714H. This supports our identification of R717L in various isolates of *Salmonella* serovars from humans and animals around the world (Fig. 6) and the emergence and spread of azithromycin resistance in *S*. Typhi and *S*. Paratyphi isolates^52^. The fact we observe this mutation to emerge rapidly and have a strong phenotypic impact on azithromycin susceptibility, which does not compromise the ability of AcrB to export other substrates when overexpressed, may make this a variant with significant benefits and helps us understand its emergence.

The Q176 residue forms part of the distal binding pocket of AcrB^9,45^, specifically participating in the so-called ‘DP_T_ cave’ structure of the pocket as defined in ref. ^63^. Due to its central position in the DP_T_ cave, Q176 has been implicated in direct binding to both substrates and non-substrates^21^ (e.g., Doxorubicin, 2DR6.pdb^9^; Rhodamine 6G^64^;), as well as competitive pump inhibitors such as D13-9001 (aka P9D) (PDB ID 3W9H^18,65^;), and pyranopyridine derivatives including MBX3135 (PDB ID 5ENR^64^), but not MBX2319. In addition, several carbapenem antibiotics have been suggested to interact directly with the Q176 based on recent MD analysis^65^, including ertapenem and biapenem. Recently, this residue has also been found in proximity to the binding site of levofloxacin (PDB ID 7B8T^66^;), further highlighting its critical role in the recognition and coordination of substrates.

Docking of cefotaxime to the WT and Q176K DBP pocket shows the side chain in direct contact with the substrate in both cases (Fig. 5, Supplementary Fig. 4). Importantly, and directly opposite to the effect of the R717L, however, the Q176K substitution seems to specifically change the binding efficiency of the DB_T_ towards cefotaxime, as the introduction of the lysine side chain produces several new strong polar contacts with the ligand, which translates to notably more favourable energy of binding and ligand recognition. A similar mechanism is inferred by nitrocefin and cephalothin docking.

Importantly, the predicted increase in pseudo binding free energy (~1.4 kcal/mol) as a result of the Q176K substitution is likely to improve recognition while keeping the affinity below an “inhibition threshold”, which would convert cefotaxime into a competitive inhibitor of the pump by increasing its residence time within the DBP^67–69^, as evidenced by previous studies involving, e.g., MBX2319 vs. minocycline binding^70^. Enhanced fitting within the DBP below the inhibition threshold thus translates into an increased probability for allosteric conformational change induced in the TM-region and/or correspondingly increased likelihood of a T- to C (O)-transition of the respective AcrB protomers^4,57,71^, resulting in more effective overall transport.

Whilst our data show that Q176K had improved recognition of cefotaxime, which translates into decreased susceptibility for strains with this change, the phenotypic impact was only evident in combination with a change in *envZ* or *ramR*. These act to either reduce drug entry through porin loss or through overexpression of *acrB* respectively. Notably, we did not identify the Q176K substitution in isolation, and it was not present in the Enterobase database. We recently characterised the role and fitness impacts of the EnvZ substitutions selected as precursors to the emergence of Q176K and found that mutation of *envZ* had a cost on biofilm formation, potentially affecting its fitness to survive in the environment and cause disease^41^. Given the likely dependence on mutation within *envZ* for the AcrB Q176K to confer a benefit, and the inability to form good biofilms, it is possible that this combination may rarely occur in nature and hence is not recorded on Enterobase.

This work has shown that using laboratory evolution can efficiently and quickly identify mutations which allow bacteria to resist important antibiotics; furthermore, this method also allows epistatic relationships to emerge and be identified. This has allowed us to identify two key changes within AcrB and also to understand their interactions with other regulators which control cellular permeability and stress responses. Importantly, we observed the probable hierarchy of selection by identifying identical mutations, emerging in the same order, in multiple lineages; for example, azithromycin resistance emerged via the initial selection of AcrB R717L, which was then accelerated by the emergence of the T18P mutation within RamR. In contrast, for cefotaxime, a change in EnvZ is the crucial first step before the Q176K AcrB substitution can exert a significant effect. The use of different conditions can also inform the possible fitness outcomes of different combinations of mutations, and we see different permissive routes to resistance in biofilm and planktonic conditions. This is important, as understanding how resistance emerges in a laboratory setting can inform selection in the real world. Our ability to model and predict emergence of resistance is an important tool in understanding AMR.

In summary, the combination of experimental evolution and mutant characterisation has demonstrated the key role of AcrB in the evolution of resistance to major antibiotics. The accumulation of additional substitutions shows how a wider network of genes controlling diverse cellular functions contributes towards increased antimicrobial tolerance. Furthermore, we show that despite similar phenotypic manifestations, the two described AcrB substitutions employ strikingly divergent molecular mechanisms, providing new insight into how this crucial bacterial defence system operates and can evolve. Understanding the potential fitness trade-offs and changes in lifestyle that are associated with resistance gain acquired via mutations in AcrB and other efflux pumps might provide value in our continuous fight against antibiotic resistance.

## Methods

### Experimental evolution model

The experimental evolution model was carried out as described in detail in ref. ^41^. Briefly, six independent *Salmonella* lineages (two exposed planktonic lineages and four exposed biofilm lineages) were exposed to 0.06 μg/ml of cefotaxime and 10 μg/ml of azithromycin, respectively. The lineages were grown in lysogeny broth (LB) with no salt at 30 °C and were serially transferred every 72 h for 17 passages. Biofilm lineages were grown on 6-mm soda lime glass beads. Cells were recovered from the beads by vortexing, and three single-cell colonies from passages 1, 9 and 17 were isolated from populations and were stored in 20% glycerol for subsequent phenotyping.

### Antimicrobial susceptibility assays

Minimum inhibitory concentrations were determined by the broth microdilution method and the agar dilution method in Mueller–Hinton broth or agar, respectively, following EUCAST guidelines^72^.

### Whole genome sequencing and analysis

Genomic DNA was normalised to 0.5 ng/µl with 10 mM Tris-HCl. Next, 0.9 µl of TD Tagment DNA Buffer (Illumina Catalogue No. 15027866) was mixed with 0.09 µl TDE1 Tagment DNA Enzyme (Illumina Catalogue No. 15027865) and 2.01 µl PCR grade water in a master mix, and 3 µl was added to a chilled 96-well plate. Then, 2 µl of normalised DNA (1 ng total) was mixed with 3 µl of the Tagmentation mix and heated to 55 °C for 10 min in a PCR block. A PCR master mix was made up using 4 µl kapa2G buffer, 0.4 µl dNTPs, 0.08 µl polymerase and 4.52 µl PCR grade water, contained in the Kap2G Robust PCR kit (Sigma Catalogue No. KK5005) per sample and 11 µl was added to each well need to be used in a 96-well plate. After this, 2 µl each of P7 and P5 of Nextera XT Index Kit v2 index primers (Illumina Catalogue No. FC-131-2001 to 2004) were added to each well. Finally, the 5 µl Tagmentation mix was added and mixed. The PCR was run at 72 °C for 3 min, 95 °C for 1 min, 14 cycles of 95 °C for 10 s, 55 °C for 20 s and 72 °C for 3 min. Following the PCR reaction, the libraries were quantified using the Quant-iT dsDNA Assay Kit, high sensitivity kit (Catalogue No. 10164582) and run on a FLUOstar Optima plate reader. Libraries were pooled following quantification in equal quantities. The final pool was double-SPRI size selected between 0.5 and 0.7X bead volumes using KAPA Pure Beads (Roche Catalogue No. 07983298001). The final pool was quantified on a Qubit 3.0 instrument and run on a High Sensitivity D1000 ScreenTape (Agilent Catalogue No. 5067-5579) using the Agilent Tapestation 4200 to calculate the final library pool molarity. The pool was run at a final concentration of 1.8 pM on an Illumina Nextseq500 instrument using a Mid Output Flowcell (NSQ® 500 Mid Output KT v2 (300 CYS) Illumina Catalogue FC-404-2003) and 15 pM on an Illumina MiSeq instrument. Illumina recommended denaturation and loading recommendations, which included a 1% PhiX spike-in (PhiX Control v3 Illumina Catalogue FC-110-3001). To determine SNPs between the parent strain and the de novo assembled *Salmonella* genomes derived from evolved isolates, Snippy version 3.1 was used (https://github.com/tseemann/snippy). *Salmonella enterica* serovar Typhimurium 14028S (accession number: CP001363) was used as the reference strain for all analyses as it is fully sequenced and annotated.

### Identification of the mutations identified in isolates from EnteroBase

The EnteroBase repository holds and curates *Salmonella* genomes, including automated annotation of all submissions and assignment of unique allele tags to annotated genes. To identify the presence of strains carrying specific mutations of interest in the database, we downloaded all the *acrB* alleles recorded. We then created a local BLAST database for each and used our mutant allele sequences to query these databases and identify alleles with 100% identity, i.e., with the substitution of interest.

### In silico modelling and antibiotic docking

*S*TmAcrB structures for ensemble docking were built as follows: (1) several homology models of the wild type, R717L, and Q176K transporters in an asymmetric LTO state were generated using the software Modeller 10.2^73,74^ and the experimental structures with the following PDB codes as templates: 2DHH, 2DR6, 2DRD, 2GIF, 2HRT, 2J8S, 3AOA, 3AOB, 3AOC, 3AOD, 3NOC, 3NOG, 3W9H, 4DX5, 4DX6, 4DX7, 4U8V, 4U8Y, 4U95, 4U96, 4ZIT, 4ZIV, 4ZJL, 5JMN, 5NC5, 5YIL, 6Q4N, 6Q4O, 6Q4P. Each pair of target and template sequences were aligned using Clustal Omega^75^. Next, 10 homology models were built for each template, using the variable target function method to perform the optimisation. Finally, the model with the highest MOLPDF was selected for the next step. (2) Ensemble docking of Azi and Cef was performed on three different groups of AcrB structures, each defined for docking the compounds to the CH2 entrance, the PBP and the DBP. The groups of structures were chosen by adapting the protocol introduced in ref. ^44^. Namely, the 29 homology model structures selected above were aligned to each of the three sites mentioned above, and the corresponding root mean square deviation (RMSDs) at those sites were calculated for each possible pair, resulting in three symmetric 29 × 29 matrices. From each matrix, we kept only the structures that exhibited global RMSD values (calculated for all the heavy atoms defining the corresponding site) larger than 1.0 Å from each other. This allowed to include a limited number of non-redundant structures displaying different conformations at the site of interest, which should improve docking accuracy^76,77^. For pairs with RMSD values below this threshold, we removed the structure with the lowest resolution from the pool. This resulted in 19 (2DHH, 2DR6, 2GIF, 2J8S, 3AOA, 3AOB, 3AOC, 3NOC, 3NOG, 3W9H, 4DX5, 4DX6, 4DX7, 4U8V, 4ZIT, 4ZJL, 5JMN, 5NC5, 6Q4P), 20 (2DHH, 2DR6, 2GIF, 2J8S, 3AOA, 3AOB, 3AOC, 3NOC, 3NOG, 3W9H, 4DX5, 4DX6, 4DX7, 4U8V, 4ZIT, 4ZJL, 5JMN, 5NC5, 5YIL, 6Q4P), and 11 (3AOB, 2DHH, 2DR6, 2GIF, 2J8S, 3AOA, 3AOC, 3NOC, 3NOG, 3W9H, 5YIL) structures used for docking ligands on the CH2 entrance, PBP and DBP, respectively. The aforementioned sites include, respectively, residues 566, 645, 649, 653, 656, 662, 676, 678, 715, 717, 719, 722, 830 (for CH2); 79, 91, 134, 135, 573, 575, 577, 617, 624, 664, 666, 667, 668, 674, 828 (for PBP); and 46, 89, 128, 130, 134, 136, 139, 176, 177, 178, 179, 180, 273, 274, 276, 277, 327, 573, 610, 612, 615, 617, 620, 628 (for DBP).

Docking was performed using the software GNINA^78^, setting the number of output poses to 10 and the remaining parameters but the exhaustiveness (128 vs. a default value of 8) to their default values. The grids were centred onto the geometrical centre of the corresponding docking site. This resulted in grids of volumes 35·25·25 Å^3^, 30·30·30 Å^3^ and 30·30·30 Å^3^ for CH2, PBP and DBP respectively.

For each ligand and each site, the top docking pose was further relaxed using AMBER20 (https://ambermd.org/AmberMD.php) and rescored with Autodock, using the AutoDock VINA scoring function implemented in GNINA to provide a qualitative estimate of the binding affinities^46^.

For single-structure docking, AutoDock VINA was used to dock compounds onto (1) *S*TmAcrB PBP, whose models were based on the L-conformers occupied by Erythromycin (PDB ID: 3AOC chain C) and Rifampicin (PDB ID: 3AOB chain C)^19^, modified to account for the M573L species-specific substitution; and (2) *S*TmAcrB DBP, whose models were derived from the T-conformer apo-structure (PDB ID: 2J8S chain B), and occupied by minocycline (PDB ID: 4DX5 chain B)^45^, modified to account for the species-specific substitutions S48T, E280K. The grid centres and volumes were the same as the ensemble docking.

### Preparation of RNA samples for qRT-PCR

RNA from biofilms was isolated using the SV Total RNA Isolation System kit (Promega). RNA was extracted from strains carrying the AcrB R717L and AcrB R717L/ RamR T18P substitutions. Biofilms of these strains were grown on the surface of lysogeny broth agar with no salt, and these were incubated for 72 h at 30 °C. Cells from each biofilm were prepared for lysis in 100 μl TE containing 50 mg/ml lysozyme and were homogenised by vortexing. RNA was isolated following the Promega kit protocol and was eluted using 100 μl of nuclease-free water. RNA quantification was performed using the Qubit RNA High Sensitivity Assay kit (Q32852).

### Quantitative reverse transcription PCR (qRT-PCR)

To determine expression levels of *acrB* and *ramA*, we performed qRT-PCR using the Luna Universal One-Step RT-qPCR Kit from NEB (E3005), using the Applied BiosystemsΤΜ 7500 Real-Time PCR system. The primers used for the qRT-PCR are listed in Supplementary Table 1. The efficiency of the primers was calculated by the generation of calibration curves for each primer pair on serially diluted DNA samples. The R2 of the calibration curves calibrated was ≥0.98 for all the primer pairs used in this study.

RNA at a final amount of 50–100 ng was added to 10 μl final volume PCR reactions, mixed with 400 nM of each primer. The cycle parameters were as follows: 10 min at 55 °C (reverse transcription step), 1-min denaturation at 95 °C and 40 cycles of 10 s at 95 °C and 1 min at 60 °C.

For each sample, two technical replicates each from two biological replicates were included (four in total) per reaction. Controls with no reverse transcriptase were also included for each RNA sample to eliminate DNA contamination.

To calculate expression levels, expression fold change was calculated using *gyrB* expression as a reference. The relative expression was determined by calculating the logarithmic base 2 of the difference between *gyrB* gene expression and target gene expression per sample.

### Drug accumulation assay

To measure changes in cellular membrane permeability to drugs, we used the resazurin accumulation assay. Strains of interest were grown to the early exponential phase (OD: 0.2–0.3) using 1:100 inoculum from an overnight culture. The cells were washed with PBS and normalised for cell density before being mixed with 10 μg/ml of resazurin in 100 μl final volume in round-bottom microtiter plates. Fluorescence was measured at 544 nm excitation and 590 nm emission in an Omega FLUOstar plate reader. Five biological replicates (with three technical replicates assayed for each) were included per strain, and resazurin-only reactions were used as controls. The assays were repeated on at least two separate occasions, with reproducible results observed each time.

### Genetic manipulations

For the gene deletion mutants, we used the λ-red gene doctoring technique as described in ref. ^79^; 300- to 400-bp-long homologous regions flanking the genes of interest were cloned into the MCS1 and MCS2 of the pDOC-K vector. The cloned regions include the first and last 10 codons of the gene to be deleted to avoid pleiotropic effects. For the *acrB* and *ramR* deletions, the upstream homologous regions were cloned *Eco*RI/ *Bam*HI in MCS1 and the downstream ones as *Xho*I/*Nhe*I in MCS2 of pDOC-K.

For the complementation of *acrB*, we used the pWKS30/AcrB plasmid previously described^80^; expression of the gene is under the control of the pBAD system and induction was achieved with the use of 0.5% (w/v) arabinose.

For the complementation of *ramR*, we used the pDOC-K/glms vector^81^. Wild-type *ramR* and ‘ramR-T18P’ alleles were cloned *Xho*I/*Hind*III in pDOC-K/glms under the control of the gene’s native promoter.

### Reporting summary

Further information on research design is available in the Nature Research Reporting Summary linked to this article.

### Supplementary information

Supplementary Material

Reporting Summary

## Data Availability

Whole genome sequencing data that support the findings of this study have been deposited in the Sequence Read Archive with the project number PRJNA529870 (accession numbers: SAMN11288384, SAMN11288382, SAMN11288381, SAMN11288380, SAMN11288379, SAMN11288378, SAMN11288370, SAMN11288368, SAMN11288366, SAMN11288361).

## References

[1] Martinez JL, Baquero F (2000). Mutation frequencies and antibiotic resistance. Antimicrob. Agents Chemother..

[2] Blair JM, Webber MA, Baylay AJ, Ogbolu DO, Piddock LJ (2015). Molecular mechanisms of antibiotic resistance. Nat. Rev. Microbiol..

[3] Piddock LJ (2006). Clinically relevant chromosomally encoded multidrug resistance efflux pumps in bacteria. Clin. Microbiol. Rev..

[4] Alav I (2021). Structure, assembly, and function of tripartite efflux and type 1 secretion systems in Gram-negative bacteria. Chem. Rev..

[5] Klenotic PA, Moseng MA, Morgan CE, Yu EW (2021). Structural and functional diversity of resistance-nodulation-cell division transporters. Chem. Rev..

[6] Li XZ, Plésiat P, Nikaido H (2015). The challenge of efflux-mediated antibiotic resistance in Gram-negative bacteria. Clin. Microbiol. Rev..

[7] Poole K (2005). Efflux-mediated antimicrobial resistance. J. Antimicrob. Chemother..

[8] Seeger MA (2006). Structural asymmetry of AcrB trimer suggests a peristaltic pump mechanism. Science.

[9] Murakami S, Nakashima R, Yamashita E, Matsumoto T, Yamaguchi A (2006). Crystal structures of a multidrug transporter reveal a functionally rotating mechanism. Nature.

[10] McNeil HE (2019). Identification of binding residues between periplasmic adapter protein (PAP) and RND efflux pumps explains PAP-pump promiscuity and roles in antimicrobial resistance. PLoS Pathog..

[11] Chen M (2022). In situ structure of the AcrAB-TolC efflux pump at subnanometer resolution. Structure.

[12] Kobayashi N, Tamura N, van Veen HW, Yamaguchi A, Murakami S (2014). β-Lactam selectivity of multidrug transporters AcrB and AcrD resides in the proximal binding pocket. J. Biol. Chem..

[13] Hobbs EC, Yin X, Paul BJ, Astarita JL, Storz G (2012). Conserved small protein associates with the multidrug efflux pump AcrB and differentially affects antibiotic resistance. Proc. Natl Acad. Sci. USA.

[14] Bohnert JA (2008). Site-directed mutagenesis reveals putative substrate binding residues in the *Escherichia coli* RND efflux pump AcrB. J. Bacteriol..

[15] Nishino K, Yamaguchi A (2001). Analysis of a complete library of putative drug transporter genes in *Escherichia coli*. J. Bacteriol..

[16] Sulavik MC (2001). Antibiotic susceptibility profiles of *Escherichia coli* strains lacking multidrug efflux pump genes. Antimicrob. Agents Chemother..

[17] Tsukagoshi N, Aono R (2000). Entry into and release of solvents by *Escherichia coli* in an organic-aqueous two-liquid-phase system and substrate specificity of the AcrAB-TolC solvent-extruding pump. J. Bacteriol..

[18] Nakashima, R. et al. Structural basis for the inhibition of bacterial multidrug exporters. *Nature* **500**, 102–106 http://www.nature.com/nature/journal/v500/n7460/abs/nature12300.html#supplementary-information (2013). 10.1038/nature12300 23812586

[19] Nakashima R, Sakurai K, Yamasaki S, Nishino K, Yamaguchi A (2011). Structures of the multidrug exporter AcrB reveal a proximal multisite drug-binding pocket. Nature.

[20] Ruggerone P, Murakami S, Pos KM, Vargiu AV (2013). RND efflux pumps: structural information translated into function and inhibition mechanisms. Curr. Top. Med. Chem..

[21] Vargiu AV, Nikaido H (2012). Multidrug binding properties of the AcrB efflux pump characterized by molecular dynamics simulations. Proc. Natl Acad. Sci. USA.

[22] Tam HK (2021). Allosteric drug transport mechanism of multidrug transporter AcrB. Nat. Commun..

[23] Alav I, Bavro VN, Blair JMA (2022). A role for the periplasmic adaptor protein AcrA in vetting substrate access to the RND efflux transporter AcrB. Sci. Rep..

[24] Schuster S, Vavra M, Kern WV (2016). Evidence of a substrate-discriminating entrance channel in the lower porter domain of the multidrug resistance efflux pump AcrB. Antimicrob. Agents Chemother..

[25] Tam HK (2020). Binding and transport of carboxylated drugs by the multidrug transporter AcrB. J. Mol. Biol..

[26] Zwama M (2018). Multiple entry pathways within the efflux transporter AcrB contribute to multidrug recognition. Nat. Commun..

[27] Kapach G (2020). Loss of the periplasmic chaperone Skp and mutations in the efflux pump AcrAB-TolC play a role in acquired resistance to antimicrobial peptides in *Salmonella typhimurium*. Front. Microbiol..

[28] Grimsey, E. M., Weston, N., Ricci, V., Stone, J. W. & Piddock, L. J. V. Overexpression of RamA, which regulates production of the multidrug resistance efflux pump AcrAB-TolC, increases mutation rate and influences drug resistance phenotype. *Antimicrob. Agents Chemother.* **64**, e02460-19 (2020). 10.1128/AAC.02460-19 PMC7179326 31988103

[29] Blair JM (2015). AcrB drug-binding pocket substitution confers clinically relevant resistance and altered substrate specificity. Proc. Natl Acad. Sci. USA.

[30] Zwama, M. & Nishino, K. Ever-adapting RND efflux pumps in Gram-negative multidrug-resistant pathogens: a race against time. *Antibiotics* **10**, 774 (2021). 10.3390/antibiotics10070774 PMC8300642 34201908

[31] Kobylka J, Kuth MS, Müller RT, Geertsma ER, Pos KM (2020). AcrB: a mean, keen, drug efflux machine. Ann. N. Y. Acad. Sci..

[32] Li XZ, Nikaido H (2009). Efflux-mediated drug resistance in bacteria: an update. Drugs.

[33] Yang, L. et al. Emergence of two AcrB substitutions conferring multidrug resistance to *Salmonella* spp. *Antimicrob. Agents Chemother.* **65**, e01589-20 (2021). 10.1128/AAC.01589-20 PMC8092907 33685897

[34] Andersson DI, Hughes D (2010). Antibiotic resistance and its cost: is it possible to reverse resistance?. Nat. Rev. Microbiol..

[35] Alav I, Sutton JM, Rahman KM (2018). Role of bacterial efflux pumps in biofilm formation. J. Antimicrob. Chemother..

[36] Piddock LJ (2006). Multidrug-resistance efflux pumps—not just for resistance. Nat. Rev. Microbiol..

[37] Costerton JW (1987). Bacterial biofilms in nature and disease. Annu. Rev. Microbiol..

[38] Trampari E (2021). Exposure of *Salmonella* biofilms to antibiotic concentrations rapidly selects resistance with collateral tradeoffs. NPJ Biofilms Microbiomes.

[39] Baucheron S (2014). Bile-mediated activation of the *acrAB* and *tolC* multidrug efflux genes occurs mainly through transcriptional derepression of *ramA* in *Salmonella enterica* serovar Typhimurium. J. Antimicrob. Chemother..

[40] Yamasaki, S. et al. The crystal structure of multidrug-resistance regulator RamR with multiple drugs. *Nat. Commun.* **4**, 2078 (2013). 10.1038/ncomms3078 23800819

[41] Trampari, E. et al. Cefotaxime exposure selects mutations within the CA-domain of *envZ* which promote antibiotic resistance but repress biofilm formation in *Salmonella*. *Microbiol. Spectr.* **10**, e0214521 (2022). 10.1128/spectrum.02145-21 PMC9241649 35475640

[42] Vidal-Aroca F, Meng A, Minz T, Page MG, Dreier J (2009). Use of resazurin to detect mefloquine as an efflux-pump inhibitor in *Pseudomonas aeruginosa* and *Escherichia coli*. J. Microbiol. Methods.

[43] Johnson, R. M. et al. Cryo-EM structure and molecular dynamics analysis of the fluoroquinolone resistant mutant of the AcrB transporter from *Salmonella*. *Microorganisms* **8**, 943 (2020). 10.3390/microorganisms8060943 PMC7355581 32585951

[44] Malvacio I (2019). Molecular basis for the different interactions of congeneric substrates with the polyspecific transporter AcrB. Biochim. Biophys. Acta Biomembr..

[45] Eicher T (2012). Transport of drugs by the multidrug transporter AcrB involves an access and a deep binding pocket that are separated by a switch-loop. Proc. Natl Acad. Sci. USA.

[46] Basciu A, Koukos PI, Malloci G, Bonvin A, Vargiu AV (2020). Coupling enhanced sampling of the apo-receptor with template-based ligand conformers selection: performance in pose prediction in the D3R Grand Challenge 4. J. Comput. Aided Mol. Des..

[47] Ababou A, Koronakis V (2016). Structures of gate loop variants of the AcrB drug efflux pump bound by erythromycin substrate. PLoS ONE.

[48] Zuo Z, Wang B, Weng J, Wang W (2015). Stepwise substrate translocation mechanism revealed by free energy calculations of doxorubicin in the multidrug transporter AcrB. Sci. Rep..

[49] Achtman M (2020). Genomic diversity of *Salmonella enterica*—the UoWUCC 10K genomes project. Wellcome Open Res..

[50] Alikhan NF, Zhou Z, Sergeant MJ, Achtman M (2018). A genomic overview of the population structure of *Salmonella*. PLoS Genet..

[51] Trampari, E. et al. Antibiotics select for novel pathways of resistance in biofilms. Preprint at *bioRxiv*10.1101/605212 (2019).

[52] Hooda Y (2019). Molecular mechanism of azithromycin resistance among typhoidal *Salmonella* strains in Bangladesh identified through passive pediatric surveillance. PLoS Negl. Trop. Dis..

[53] Scribner, M. R., Santos-Lopez, A., Marshall, C. W., Deitrick, C. & Cooper, V. S. Parallel evolution of tobramycin resistance across species and environments. *mBio* **11**, e00932-20 (2020). 10.1128/mBio.00932-20 PMC7251211 32457248

[54] Li Y, Cross TS, Dörr T (2022). Analysis of AcrB in *Klebsiella pneumoniae* reveals natural variants promoting enhanced multidrug resistance. Res. Microbiol..

[55] Yao, H. et al. Emergence of a potent multidrug efflux pump variant that enhances *Campylobacter* resistance to multiple antibiotics. *mBio* **7**, e01543-16 (2016). 10.1128/mBio.01543-16 PMC5030363 27651364

[56] Yu EW, Aires JR, McDermott G, Nikaido H (2005). A periplasmic drug-binding site of the AcrB multidrug efflux pump: a crystallographic and site-directed mutagenesis study. J. Bacteriol..

[57] Eicher, T. et al. Coupling of remote alternating-access transport mechanisms for protons and substrates in the multidrug efflux pump AcrB. *Elife* **3**, e03145 (2014). 10.7554/eLife.03145 PMC4359379 25248080

[58] Reading E (2020). Perturbed structural dynamics underlie inhibition and altered efflux of the multidrug resistance pump AcrB. Nat. Commun..

[59] Vargiu AV (2018). Water-mediated interactions enable smooth substrate transport in a bacterial efflux pump. Biochim. Biophys. Acta Gen. Subj..

[60] Zwama M, Nishino K (2022). Proximal binding pocket Arg717 substitutions in *Escherichia coli* AcrB cause clinically relevant divergencies in resistance profiles. Antimicrob. Agents Chemother..

[61] Lyu, M. et al. Cryo-EM structures of a gonococcal multidrug efflux pump illuminate a mechanism of drug recognition and resistance. *mBio* **11**, e00996-20 (2020). 10.1128/mBio.00996-20 PMC7251214 32457251

[62] Ma, K. C., Mortimer, T. D. & Grad, Y. H. Efflux pump antibiotic binding site mutations are associated with azithromycin nonsusceptibility in clinical *Neisseria gonorrhoeae* isolates. *mBio* **11**, e01509-20 (2020). 10.1128/mBio.01509-20 PMC7448274 32843551

[63] Takatsuka Y, Chen C, Nikaido H (2010). Mechanism of recognition of compounds of diverse structures by the multidrug efflux pump AcrB of *Escherichia coli*. Proc. Natl Acad. Sci. USA.

[64] Sjuts H (2016). Molecular basis for inhibition of AcrB multidrug efflux pump by novel and powerful pyranopyridine derivatives. Proc. Natl Acad. Sci. USA.

[65] Atzori, A. et al. Molecular interactions of carbapenem antibiotics with the multidrug efflux transporter AcrB of *Escherichia coli*. *Int. J. Mol. Sci*. **21**, 860 (2020). 10.3390/ijms21030860 PMC7037162 32013182

[66] Ornik-Cha A (2021). Structural and functional analysis of the promiscuous AcrB and AdeB efflux pumps suggests different drug binding mechanisms. Nat. Commun..

[67] Dey, D., Kavanaugh, L. G. & Conn, G. L. Antibiotic substrate selectivity of *Pseudomonas aeruginosa* MexY and MexB efflux systems is determined by a Goldilocks affinity. *Antimicrob. Agents Chemother.* **64**, e00496-20 (2020). 10.1128/AAC.00496-20 PMC7526836 32457110

[68] Kinana AD, Vargiu AV, Nikaido H (2016). Effect of site-directed mutations in multidrug efflux pump AcrB examined by quantitative efflux assays. Biochem. Biophys. Res. Commun..

[69] Vargiu AV (2011). Effect of the F610A mutation on substrate extrusion in the AcrB transporter: explanation and rationale by molecular dynamics simulations. J. Am. Chem. Soc..

[70] Vargiu AV, Ruggerone P, Opperman TJ, Nguyen ST, Nikaido H (2014). Molecular mechanism of MBX2319 inhibition of *Escherichia coli* AcrB multidrug efflux pump and comparison with other inhibitors. Antimicrob. Agents Chemother..

[71] Glavier M (2020). Antibiotic export by MexB multidrug efflux transporter is allosterically controlled by a MexA-OprM chaperone-like complex. Nat. Commun..

[72] European Committee on Antimicrobial Susceptibility Testing (EUCAST). https://www.eucast.org/ (2016).

[73] Grimsey, E. M. et al. Chlorpromazine and amitriptyline are substrates and inhibitors of the AcrB multidrug efflux pump. *mBio* **11**, e00465-20 (2020). 10.1128/mBio.00465-20 PMC7267879 32487753

[74] Webb, B. & Sali, A. Comparative protein structure modeling using MODELLER. *Curr. Protoc. Bioinformatics* **54**, 5.6.1–5.6.37 (2016). 10.1002/cpbi.3 PMC5031415 27322406

[75] Sievers F (2011). Fast, scalable generation of high-quality protein multiple sequence alignments using Clustal Omega. Mol. Syst. Biol..

[76] Asthana S, Shukla S, Ruggerone P, Vargiu AV (2014). Molecular mechanism of viral resistance to a potent non-nucleoside inhibitor unveiled by molecular simulations. Biochemistry.

[77] Basciu A, Malloci G, Pietrucci F, Bonvin A, Vargiu AV (2019). Holo-like and druggable protein conformations from enhanced sampling of binding pocket volume and shape. J. Chem. Inf. Model..

[78] McNutt AT (2021). GNINA 1.0: molecular docking with deep learning. J. Cheminform..

[79] Lee DJ (2009). Gene doctoring: a method for recombineering in laboratory and pathogenic *Escherichia coli* strains. BMC Microbiol..

[80] Baugh S, Ekanayaka AS, Piddock LJ, Webber MA (2012). Loss of or inhibition of all multidrug resistance efflux pumps of *Salmonella enterica* serovar Typhimurium results in impaired ability to form a biofilm. J. Antimicrob. Chemother..

[81] Holden ER, Wickham GJ, Webber MA, Thomson NM, Trampari E (2020). Donor plasmids for phenotypically neutral chromosomal gene insertions in Enterobacteriaceae. Microbiology.

