# Supplementary material for: Functionally distinct mutations within AcrB underpin antibiotic resistance in different lifestyles
Source: NPJ Antimicrob Resist. 2023 May 10;1:2. doi: 10.1038/s44259-023-00001-8 (PMC11057200; doi:10.1038/s44259-023-00001-8)
Supplement: Supplementary file 1 — Supplementary Material [file 44259_2023_1_MOESM1_ESM.pdf]

Supplementary materials

Supplementary Table 1. Primers used in this study

Name	Sequence	Use
AcrB-part1 for (EcoRI)	TACGTGAATTCCACGCGGCGATGCCACGGTG	*acrB* deletion
AcrB-part1 Rev (BamHI)	GCATAGGATCCAAATATAGGGCGATCGATAA	*acrB* deletion
AcrB-part2 for (XhoI)	TACGTCTCGAGATTGAGCATAGTCATTCGAC	*acrB* deletion
AcrB-part2 Rev (NheI)	GCATAGCTAGCGTTTGTGTAATCATTGGGTT	*acrB* deletion
RamR part1-For (EcoRI)	TACGTGAATTCAAACTCGTCAGCGGCTCCCG	*ramR* deletion
RamR-part1 Rev (BamHI)	GCATAGGATCCTTTTTTGTCTTCACTCTTCG	*ramR* deletion
RamR-part2 for (XhoI)	TACGTCTCGAGTGGCGCGCGCTGACTCGCGA	*ramR* deletion
RamR-part2 Rev (NheI)	GCATAGCTAGCTATCCTCGCCCGCATAGACT	*ramR* deletion
RamR-compl-For (XhoI)	GCATACTCGAGTTCATGCGGCAGCCCTTG	*ramR* complementation
RamR-compl-Rev (HindIII)	ACGTAAAGCTTTTATTGCTCCTCGCGAGTCAGC	*ramR* complementation
GyrB-RT-For	GGAAGGGGACTCCGCGGGCG	q-RT PCR
GyrB-RT-Rev	CAGCGGCGGCTGCGCAATGT	q-RT PCR
RamA-RT-For	CGCTCAGGTTATCGACACGA	q-RT PCR
RamA-RT-Rev	CCACTTGGAATACCCCGCAT	q-RT PCR

Supplementary Figure 1. Emergence of resistance

A

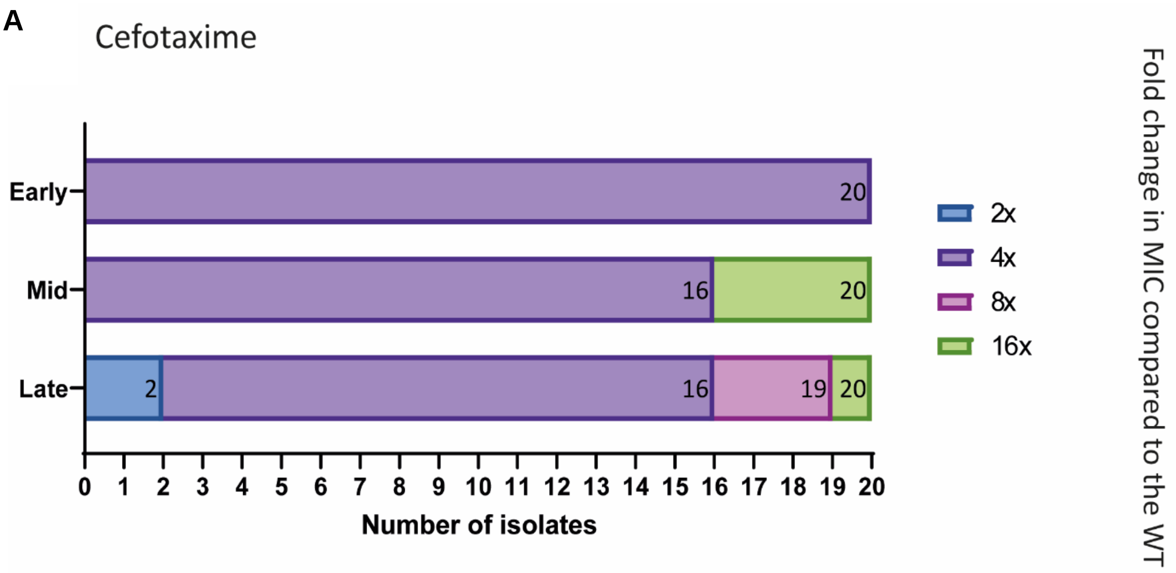

B

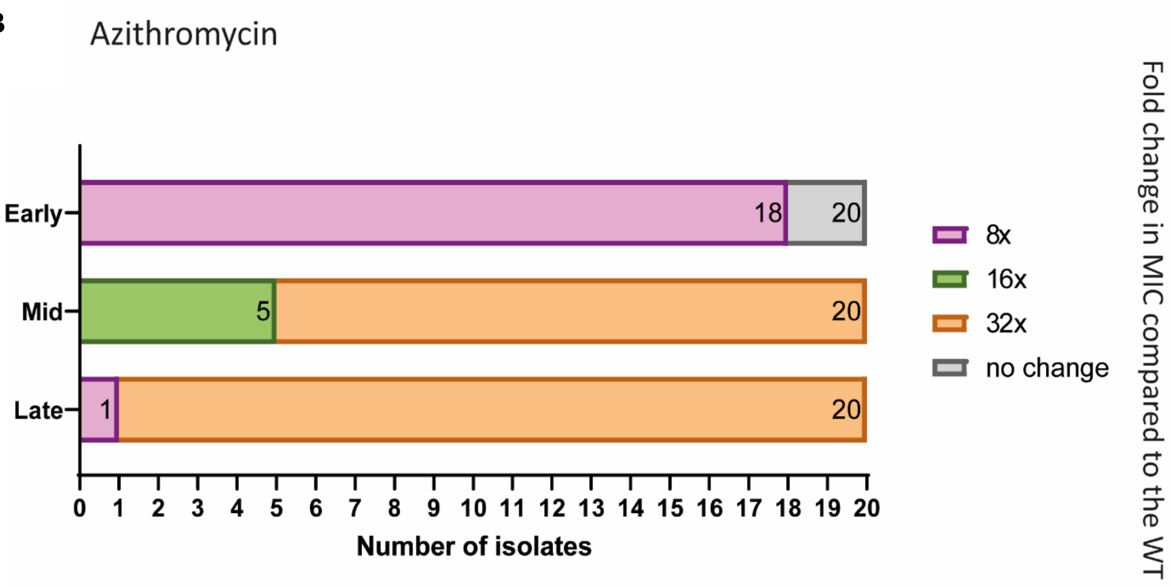

Stacked bars showing how many isolates (out of the 20 phenotyped in total), had 2, 4, 8, 16, 32 fold changes in MIC compared to the WT. For cefotaxime, the WT MIC was 0.125 µg/ mL and 4 µg/ mL for azithromycin. **A.** All strains exposed to cefotaxime, exhibited a 4x change in MIC at the earliest sampling timepoint. In the middle of the experiment (passage 9), we isolated 4 with a 16x increase. At the latest timepoint, there was more variation with isolates MICs being 2x to 16x higher. B. Under azithromycin exposure, we isolated 18 strains with an MIC 8x higher that the WT and 2 sharing the same MIC as the WT. In the middle of the experiment (passage 9), 5 strains had an MIC 16x higher than the WT and 15 showed a 32x increase. At the end of the experiment, 1 showed an 8x increase and 19 of the isolates exhibited a 32 fold change.

Supplementary Figure 2. Fitness of selected mutants.

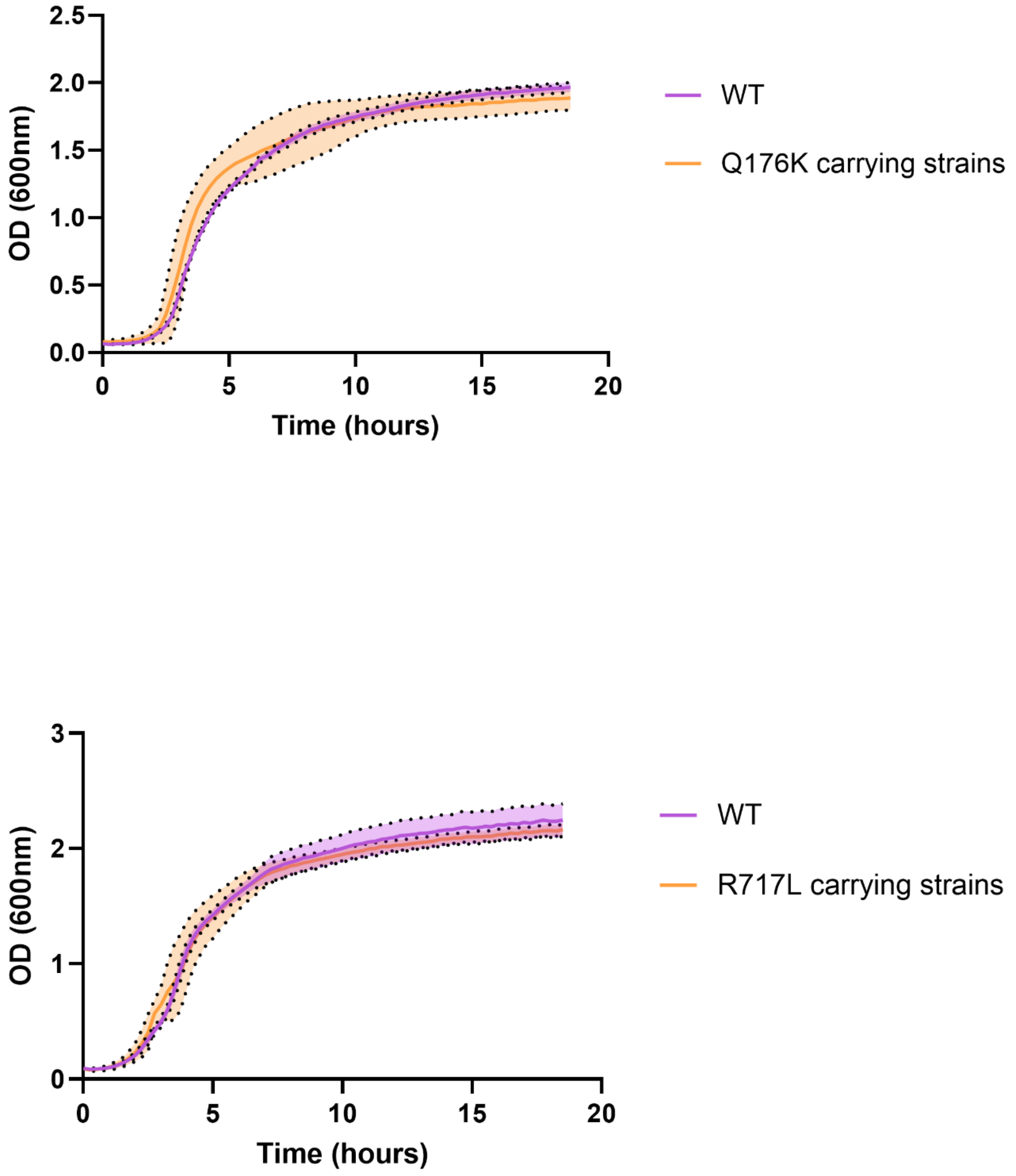

Growth curves for 3 independent isolates carrying the Q176H and R717L mutations within AcrB, demonstrating that no significant changes in growth were observed compared to the WT when grown in LB at 37ºC. Lines represent the mean OD and the shaded areas around the curves indicate standard errors.

Supplementary Figure 3. Distribution of docking poses of azithromycin to CH2 and PBP in WT and R717L.

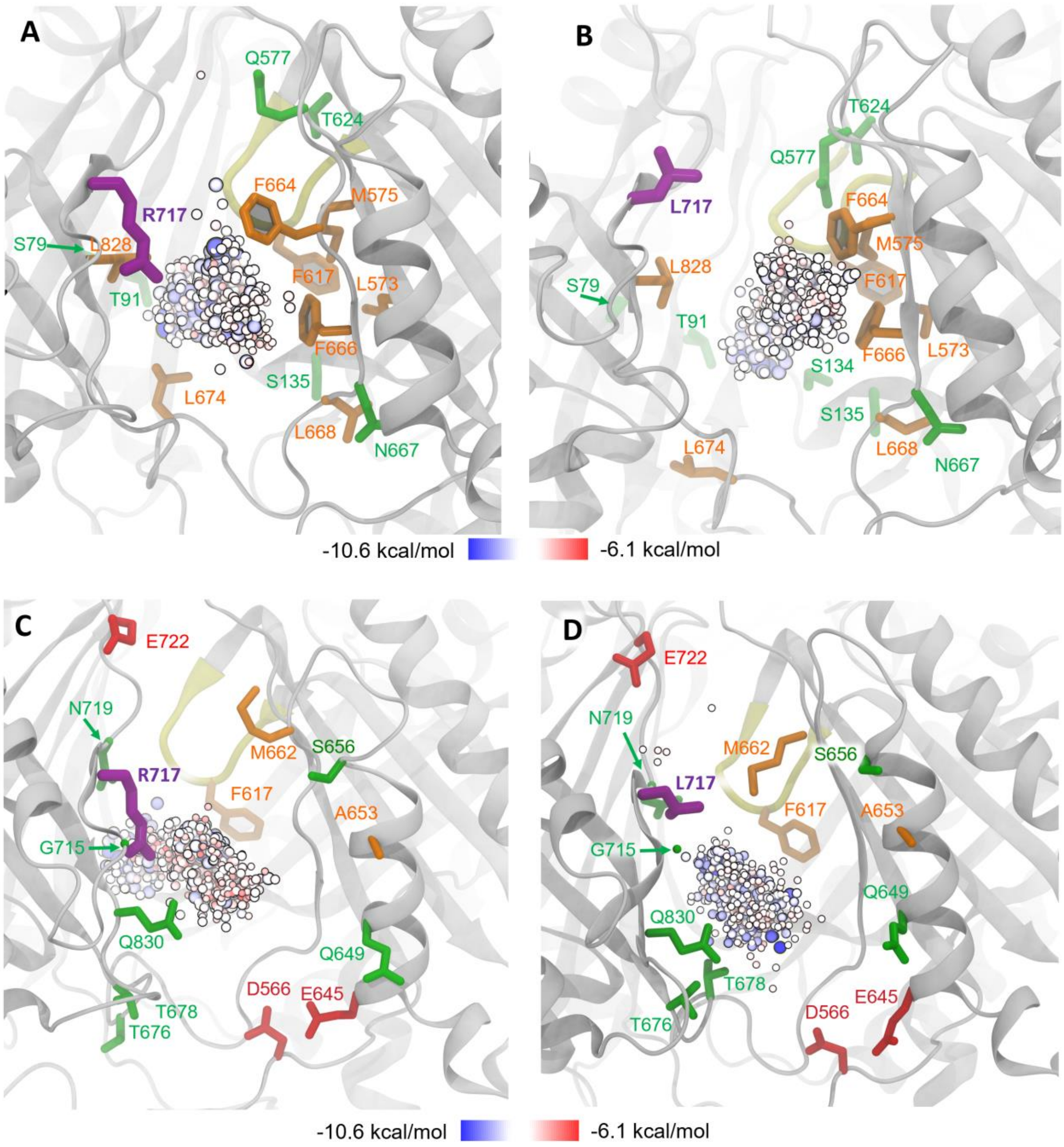

Distribution of the docking poses for Azi obtained for the WT and R717L AcrB protein to the CH2 and PBP regions. WT protein is shown in **A** (CH2) and **C** (PBP)**,** and the R717L variant in **B** and **D.** The centres of the poses for Azi are represented by spheres coloured and sized according to their affinity (increasing from red to white to blue, and by the sphere radius). The G-loop separating the PBP from DBP is coloured olive green, with PBP facing the viewer. Sidechains of residues participating in ligand binding are shown as sticks.

Supplementary Figure 4. Distribution of docking poses of cefotaxime to the DBP in WT and Q176K.

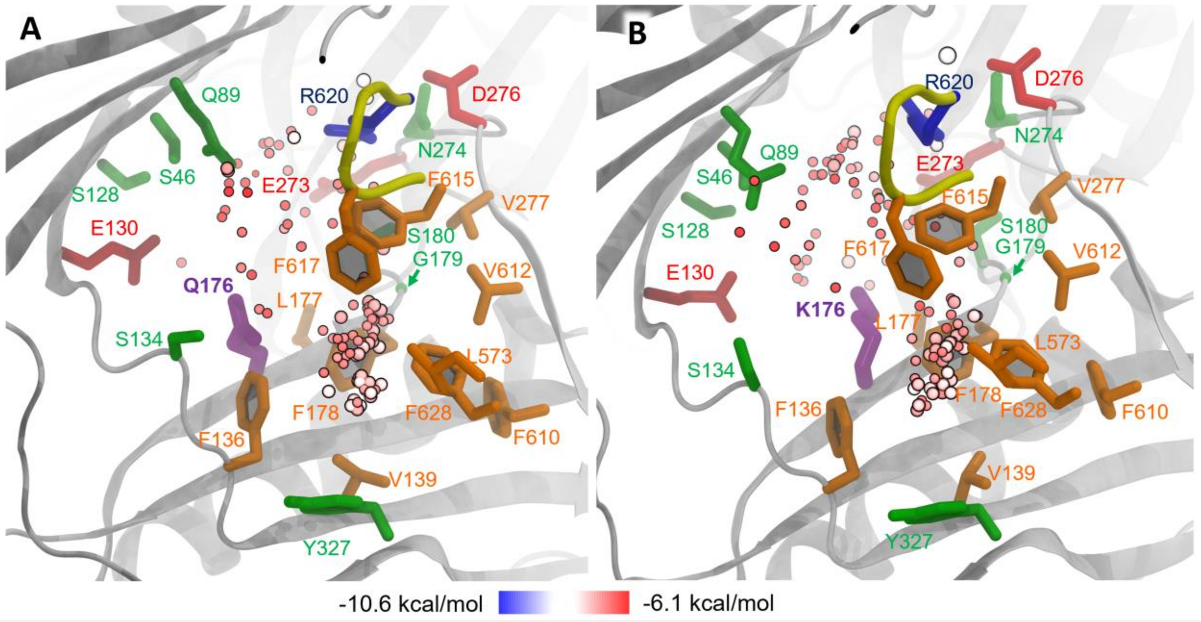

A. Distribution of the docking poses for cefotaxime in the wild type DBP. **B.** Distribution of the docking poses of cefotaxime in the Q176K pocket. Spheres coloured as in supplementary figure 1.
