# Supplementary material for: Functionally distinct mutations within AcrB underpin antibiotic resistance in different lifestyles
Source: NPJ Antimicrob Resist. 2023 May 10;1:2. doi: 10.1038/s44259-023-00001-8 (PMC11057200; doi:10.1038/s44259-023-00001-8)
Supplement: Supplementary file 2 — Reporting Summary [file 44259_2023_1_MOESM2_ESM.pdf]

nature research

Corresponding author(s): Mark Webber

Last updated by author(s): Feb 2, 2023

Reporting Summary

Nature Research wishes to improve the reproducibility of the work that we publish. This form provides structure for consistency and transparency in reporting. For further information on Nature Research policies, see Authors & Referees and the Editorial Policy Checklist.

Statistics

For all statistical analyses, confirm that the following items are present in the figure legend, table legend, main text, or Methods section.

n/a	Confirmed	
☐	☒	The exact sample size (n) for each experimental group/condition, given as a discrete number and unit of measurement
☐	☒	A statement on whether measurements were taken from distinct samples or whether the same sample was measured repeatedly
☐	☒	The statistical test(s) used AND whether they are one- or two-sided<br>*Only common tests should be described solely by name; describe more complex techniques in the Methods section.*
☒	☐	A description of all covariates tested
☐	☒	A description of any assumptions or corrections, such as tests of normality and adjustment for multiple comparisons
☐	☒	A full description of the statistical parameters including central tendency (e.g. means) or other basic estimates (e.g. regression coefficient) AND variation (e.g. standard deviation) or associated estimates of uncertainty (e.g. confidence intervals)
☒	☐	For null hypothesis testing, the test statistic (e.g. F, t, r) with confidence intervals, effect sizes, degrees of freedom and P value noted<br>*Give P values as exact values whenever suitable.*
☒	☐	For Bayesian analysis, information on the choice of priors and Markov chain Monte Carlo settings
☒	☐	For hierarchical and complex designs, identification of the appropriate level for tests and full reporting of outcomes
☒	☐	Estimates of effect sizes (e.g. Cohen's d, Pearson's r), indicating how they were calculated

Our web collection on statistics for biologists contains articles on many of the points above.

Software and code

Policy information about availability of computer code

Data collection	All software and versions used are described in the text. All software is freely available.
Data analysis	All software and versions used are described in the text. All software is freely available.

For manuscripts utilizing custom algorithms or software that are central to the research but not yet described in published literature, software must be made available to editors/reviewers. We strongly encourage code deposition in a community repository (e.g. GitHub). See the Nature Research guidelines for submitting code & software for further information.

Data

Policy information about availability of data

All manuscripts must include a data availability statement. This statement should provide the following information, where applicable:

- Accession codes, unique identifiers, or web links for publicly available datasets
- A list of figures that have associated raw data
- A description of any restrictions on data availability

Whole genome sequencing data that support the findings of this study have been deposited in the Sequence Read Archive with the project number PRJNA529870. All other data that support findings of this study are available from the corresponding author upon reasonable request.

Field-specific reporting

Please select the one below that is the best fit for your research. If you are not sure, read the appropriate sections before making your selection.

☐ Life sciences ☐ Behavioural & social sciences ☒ Ecological, evolutionary & environmental sciences

Ecological, evolutionary & environmental sciences study design

All studies must disclose on these points even when the disclosure is negative.

Study description	In vitro evolution study of bacteria exposed to different drug stresses (azithromycin, cefotaxime). Analysis of genomes of selected mutants and characterisation of mutants phenotypically and in silico modelling of mutant protein (efflux pump) structures. Shows mutant efflux pumps are key to antibiotic resistance but different mutations work in different ways
Research sample	Experiments all used Salmonella enterica serovar Typhimurium 14028S as a parent stain for evolution experiments. Populations were recovered after drug exposures and stored before being phenotyped.
Sampling strategy	After each drug exposure cycle all cells were recovered and the population stored from all lineages
Data collection	Data was collected by researchers working on the project and recorded in electronic lab books which are archived indefinitely
Timing and spatial scale	Each passage lasted 72 hours to allow biofilm formation and samples were collected after each passage was completed from all lineages in each experiment
Data exclusions	No data was excluded
Reproducibility	All experiments included multiple independent lineages and results from each were compared
Randomization	All lineages were initiated with independent cultures of the same starting strain, these were randomly allocated
Blinding	For phenotypic testing, scientists were blinded to which lineage was being tested

Did the study involve field work? ☐ Yes ☒ No

Reporting for specific materials, systems and methods

We require information from authors about some types of materials, experimental systems and methods used in many studies. Here, indicate whether each material, system or method listed is relevant to your study. If you are not sure if a list item applies to your research, read the appropriate section before selecting a response.

Materials & experimental systems

n/a	Involved in the study	
☒	☐	Antibodies
☒	☐	Eukaryotic cell lines
☒	☐	Palaeontology
☒	☐	Animals and other organisms
☒	☐	Human research participants
☒	☐	Clinical data

Methods

n/a	Involved in the study	
☒	☐	ChIP-seq
☒	☐	Flow cytometry
☒	☐	MRI-based neuroimaging
